# Planar and Curved π-Extended Porphyrins by On-Surface Cyclodehydrogenation

**DOI:** 10.1021/jacs.4c12460

**Published:** 2024-12-04

**Authors:** Miloš Baljozović, Joffrey Pijeat, Stéphane Campidelli, Karl-Heinz Ernst

**Affiliations:** † Molecular Surface Science Group, Empa, 8600 Dübendorf, Switzerland; ‡ Université Paris-Saclay, CEA, CNRS, NIMBE, LICSEN, 91191 Gif-sur-Yvette, France; § Department of Chemistry, University of Zürich, 8057 Zürich, Switzerland; ∥ Nanosurf Laboratory, Institute of Physics, The Czech Academy of Sciences, 16200 Prague, Czech Republic

## Abstract

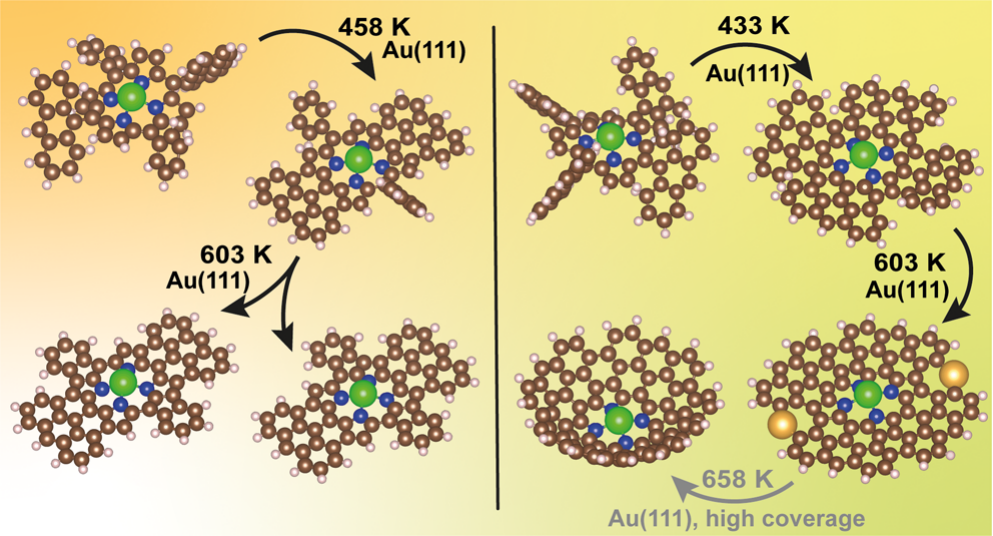

Recent advancements in on-surface synthesis have enabled the reliable and predictable preparation of atomically precise low-dimensional materials with remarkable properties, which are often unattainable through traditional wet chemistry. Among these materials, porphyrins stand out as a particularly intriguing class of molecules, extensively studied both in solution and on surfaces. Their appeal lies in the ability to fine-tune their unique chemical and physical properties through central metal exchange or peripheral functionalization. However, the synthesis of π-extended porphyrins featuring unsubstituted anthracenyl groups has remained elusive. Herein, we report an *in vacuo* temperature-controlled cyclodehydrogenation of bis- and tetraanthracenyl Zn(II) porphyrins on a gold(111) surface. By gradually increasing the temperature, sequential dehydrogenation leads to the formation of fused anthracenyl porphyrin products. Notably, at high molecular coverage, the formation of bowl-shaped porphyrins occurs, along with transmetalation of Zn with Au. These findings open the door to a variety of π-extended anthracenyl-containing porphyrin products via cyclodehydrogenation and transmetalation, offering significant potential in the fields of molecular (photo/electro)catalysis, (opto)electronics, and spintronics.

## Introduction

Porphyrins are tetrapyrrolic aromatic macrocycles with pyrrolic moieties connected via four methinebridges. The cavity of the macrocycle has the ability to host metals, and metalated porphyrins are building blocks of natural systems such as heme and chlorophyll, playing a paramount biological role in oxygen transport, light harvesting, and numerous redox processes.^1,2^ Synthetic analogues of naturally occurring macrocycles have been the focus of a vast number of research areas spanning from biological model systems to components of electronic, spintronic, optoelectronic, and photovoltaic devices.^3−10^ Another important aspect of research on porphyrins concerns the investigation of their chemical and physical properties on surfaces.^11−20^ Moreover, for metalated macrocycles in a curved aromatic sheet, exceptional electrocatalytic properties for water splitting are expected.^21^

The chemical and physical properties of porphyrins can be precisely controlled through central metal modification or peripheral functionalization.^22−25^ The latter, in particular, has been shown to depend heavily on both the number and nature of peripheral functional groups, which are crucial for fine-tuning these properties.^23,25,26^ Especially noteworthy is functionalization that involves oxidative coupling (fusion) of polycyclic aromatic hydrocarbons attached in the *meso* position of porphyrin to the β positions of the pyrrolic subunits. Such process expands the aromatic system, thereby significantly improving the optical properties of the porphyrin.^26−38^

Among the polyaromatic hydrocarbons to be fused to porphyrins, anthracene stands out as particularly elegant when attached at the *meso* position. The C1 and C8 carbon atoms of anthracene can form two new C–C bonds with the β-pyrrolic carbon atoms of porphyrin, creating a highly integrated and structurally refined system.

**Scheme 1:**
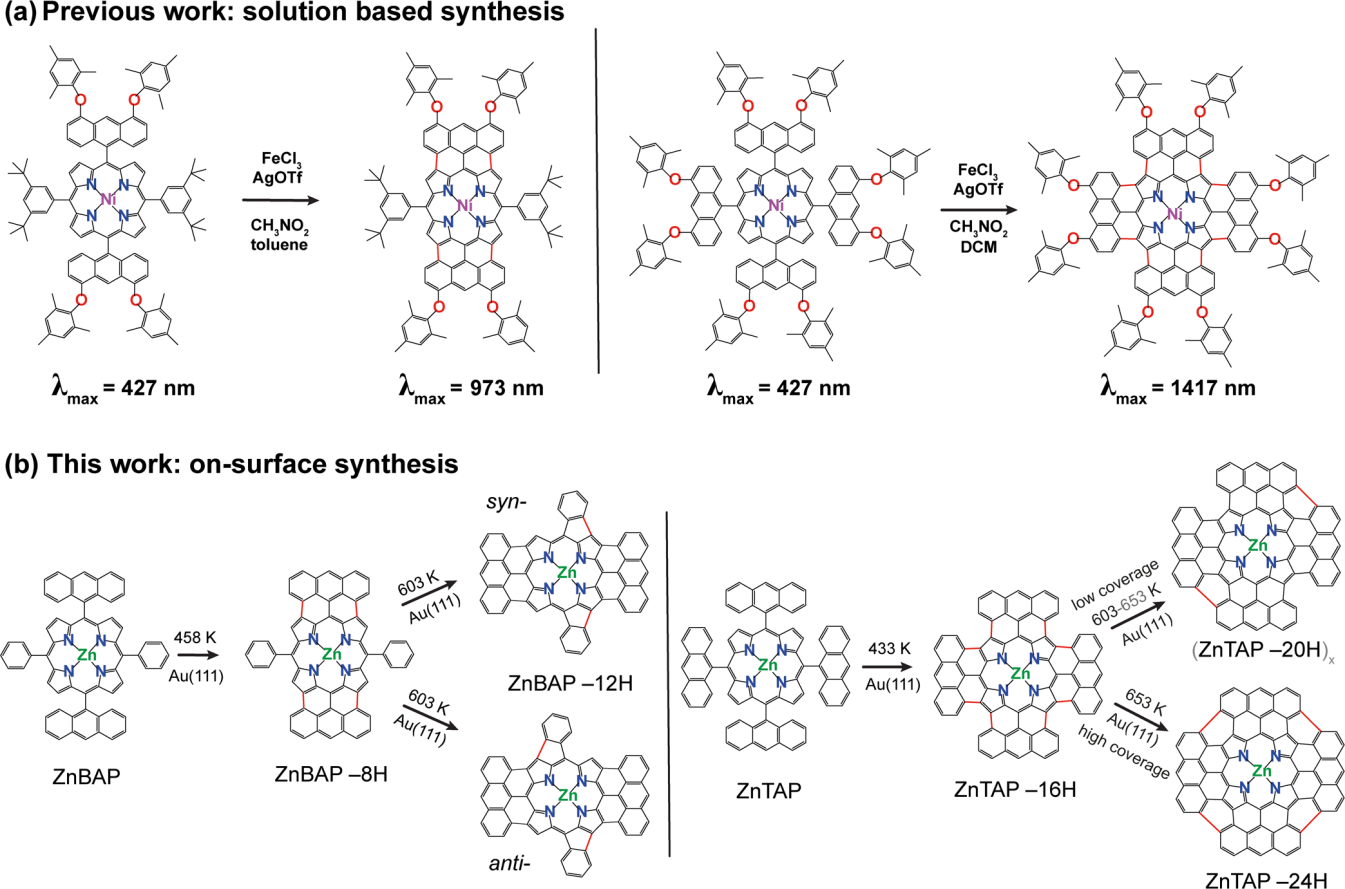
Comparison of Solution and On-Surface Synthesis of Fused Anthracenyl Porphyrins

About a decade ago, Anderson and co-workers reported the synthesis of fused bis- and tetraanthracenyl Ni(II) porphyrins, showcasing remarkable shifts in absorbance maxima upon fusion (Scheme 1a).^27,28^ However, the formation of π-extended porphyrins bearing unsubstituted anthracenyl moieties or overoxidized anthracenyl porphyrin derivatives has yet to be achieved. Herein, we demonstrate the formation of such compounds by an on-surface synthesis method (Scheme 1b). On-surface synthesis has recently become a leading technique for controllable C–C bond formation that would otherwise hardly be accessible by conventional solution chemistry. It enables the reliable and predictable preparation of atomically precise low-dimensional materials with unprecedented properties, identified and characterized primarily through scanning probe techniques.^39−47^

The temperature-controlled synthesis of fused Zn bis- and tetraanthracen-9-yl porphyrins (**ZnBAP** and **ZnTAP**) proceeds by cyclodehydrogenation of the corresponding *meso*-substituted porphyrins on the Au(111) surface. Notably, on-surface dehydrogenation is not limited to the first dehydrogenation step involving the fusion of anthracenyl units to the macrocycle. Sequential dehydrogenation with temperature elevation leads to new products, as identified by scanning tunneling microscopy (STM), time-of-flight secondary ion mass spectrometry (ToF-SIMS), and X-ray photoemission spectroscopy (XPS).

## Results and Discussion

### Synthesis of Porphyrin Derivatives

**ZnBAP** and **ZnTAP** were synthesized according to procedures derived from the literature^48,49^ (see the Experimental Section and Supporting Information for details).

### ToF-SIMS Analysis of Surface Reaction Products

**Figure 1:**
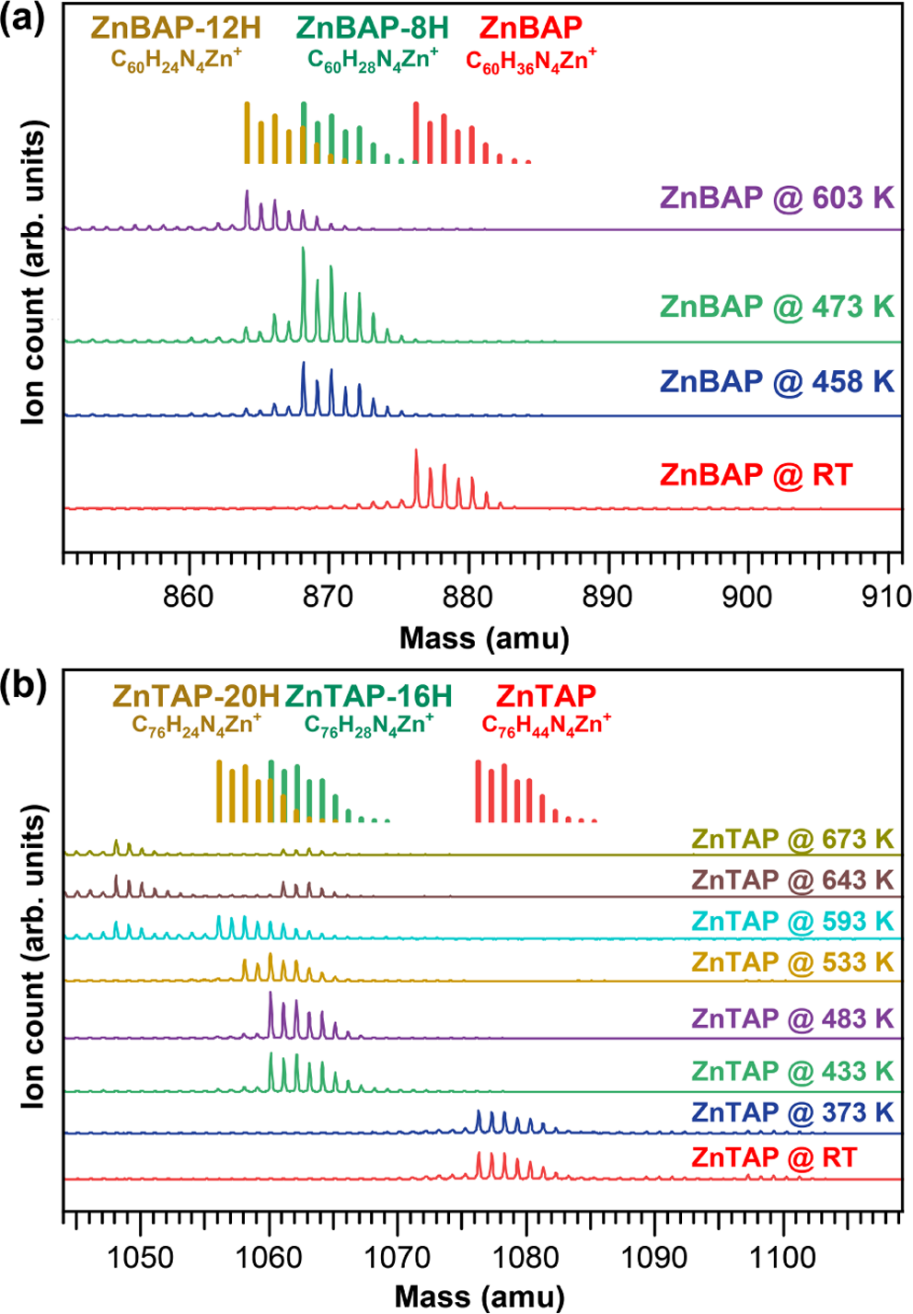
ToF-SIM spectra tracking mass loss due to cyclodehydrogenation of Zn-anthracenyl porphyrins upon thermal activation. (a) Temperature series of **ZnBAP** on Au(111) leading to **ZnBAP-8H** and **ZnBAP-12H** products upon annealing to 458 and 603 K, respectively. Colored bars above the spectra represent expected mass distributions of C_60_H_36_N_4_Zn^+^ (**ZnBAP**), C_60_H_28_N_4_Zn^+^ (**ZnBAP-8H**), and C_60_H_24_N_4_Zn^+^ (**ZnBAP-12H**) ions. (b) ToF-SIMS temperature series of **ZnTAP** on Au(111) showing masses corresponding to **ZnTAP**, **ZnTAP-16H**, and **ZnTAP-20H**. Simulated mass distributions thereof are shown in the spectra.

After deposition of **ZnBAP** and **ZnTAP** onto the clean Au(111) surface, stepwise annealing led to fused anthracenyl porphyrins. The results are summarized in Figure 1.

Upon deposition, **ZnBAP** is identified by the signal appearing from 876.2 to 884.2 amu with its characteristic isotopic pattern that corresponds to the C_60_H_36_N_4_Zn^+^ (ZnBAP^+^) ion. Upon annealing to 458 K, the signal shifts by 8 amu downward, corresponding to the loss of 8 H atoms and formation of 4 single C–C bonds (**ZnBAP-8H**). This loss is ascribed to the on-surface cyclodehydrogenation-induced fusion of both anthracenyl units at the β-pyrrolic carbon positions of porphyrin (Scheme 1b, left). The fusion of two anthracenyl units occurs at a temperature that is significantly lower than the temperature needed for cyclodehydrogenation of bisanthracene oligomers or bisanthracene–porphyrin hybrids,^50−52^ however, well in line with the temperature required for cyclodehydrogenation of 5,15-bis(10-bromoanthracen-9-yl)-10,20-bis(trifluoromethyl)porphyrins on Au(111).^53^ The reason for the reduction here is likely due to the improved mobility of gold adatoms and their better access to the reaction centers due to the absence of halogens on the surface as well as amenable molecular conformation. Gold adatoms have lately been identified as catalysts for on-surface (cyclo)dehydrogenation.^54−58^ Further annealing to 603 K leads to the additional signal shift by 4 amu due to fusion of the phenyl groups of the **ZnBAP-8H** molecule. The fusion can lead to both *anti-* and *syn-***ZnBAP-12H** species, as indicated in Scheme 1b, left. Notably, such products were not observed in the previous work, namely, the solution-based synthesis.^28^

**ZnTAP** molecules are identified in ToF-SIMS (Figure 1b) by their characteristic mass signals appearing from 1076.3 to 1086.3 amu. At 433 K, a 16 amu shift toward lower mass is observed, indicating a loss of 16 H atoms (**ZnTAP-16H**). Such loss corresponds to the formation of 8 C–C single bonds due to fusion of all four anthracenyl units to the porphyrin macrocycle (Scheme 1b, right). This temperature is almost identical to the temperature when anthracenyl units fuse to the macrocycle in the case of **ZnBAP**. A further loss of an additional 4 amu at 593 K suggests the formation of additional C–C bonds between adjacent anthracenyl units (**ZnTAP-20H**, Scheme 1b, right). Note that this product was also not observed in solution-based work.^27^ Annealing above 593 K leads to intermolecular coupling/polymerization as well as transmetalation with Au (*vide infra*), thus inducing the loss of specific signals in ToF-SIMS.

### STM of Surface Products

**Figure 2:**
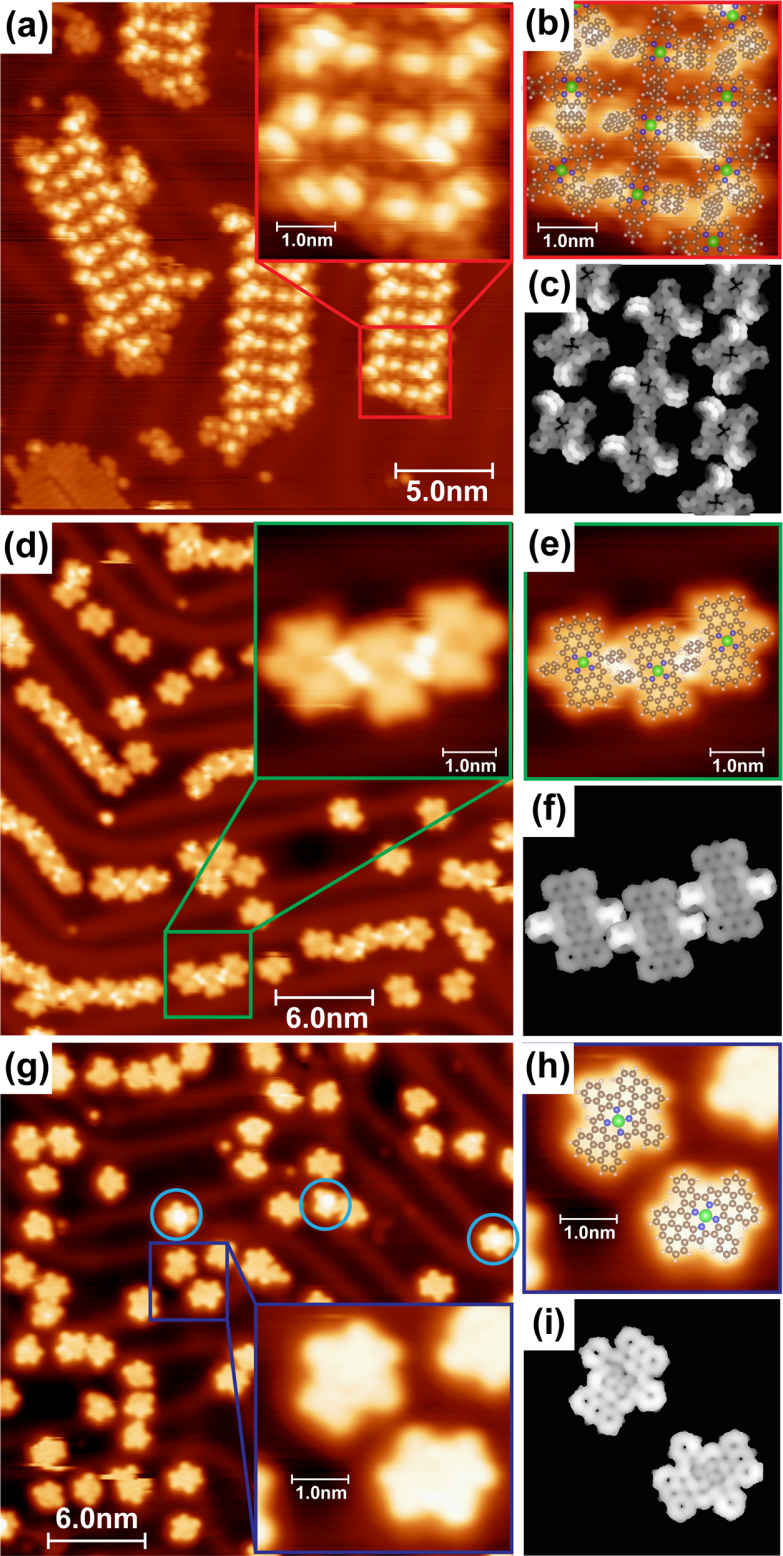
STM identification of native and cyclodehydrogenated **ZnBAP** molecular species. (a) Overview STM image (25 × 25 nm^2^) of native **ZnBAP** molecules on Au(111) assembled into braid-like structures. (b) Close-up STM image (5 × 5 nm^2^) from (a) overlaid with ball-and-stick molecular models. (c) Simulated unoccupied state density maps of **ZnBAP** molecules as arranged in (b). (d) Overview STM image (30 × 30 nm^2^) of **ZnBAP-8H** molecules on Au(111) obtained after being annealed to 468 K. (e) Close-up STM image (5 × 5 nm^2^) of three **ZnBAP-8H** molecules overlaid with molecular models. (f) Simulated occupied state density maps of **ZnBAP-8H** molecules from (e). (g) Overview STM image (30 × 30 nm^2^) of **ZnBAP-12H** molecules on Au(111) obtained after annealing to 603 K. The molecules encircled in blue appear much brighter due to transmetalation of Zn with Au. (h) Close-up STM image (5 × 5 nm^2^) of two **ZnBAP-12H** molecules overlaid with the molecular models. (i) Simulated occupied state density maps of *anti-* and *syn-***ZnBAP-12H** molecules from (h).

**Figure 3:**
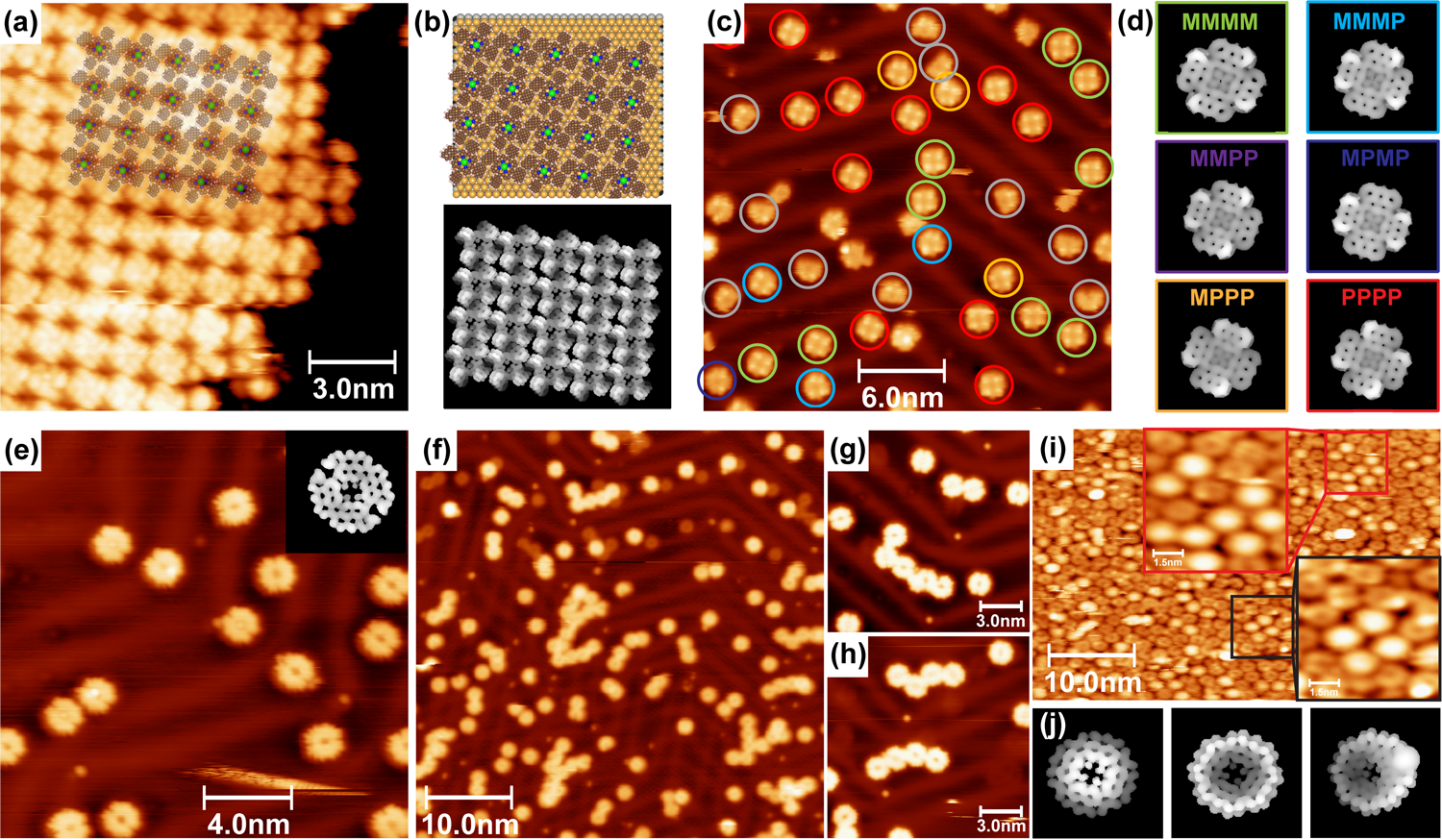
STM identification of native and cyclodehydrogenated **ZnTAP** molecular species. (a) Overview STM image (15 × 15 nm^2^) of native **ZnTAP** molecules self-assembled on Au(111). The image is superimposed with ball-and-stick molecular models. (b) Ball-and-stick molecular model of the molecular assembly from (a), together with simulated unoccupied state density maps of **ZnTAP** molecules as arranged in the model. (c) Overview STM image (30 × 30 nm^2^) of **ZnTAP-16H** molecules imaged after annealing to 483 K. Molecules are encircled depending on the conformation with the color code from (d). Gray encircled molecules appear as if they are missing one anthracenyl unit, an impurity from the synthesis or fragmentation during the sublimation. (d) Simulated occupied state density maps of six possible conformations of **ZnTAP-16H** molecules. The labeling of the conformers is performed starting from the upper left corner. (e) Overview STM image (20 × 20 nm^2^) of **ZnTAP-20H** molecules imaged after annealing to 613 K. The inset represents simulated unoccupied state density map of an adatom-coordinated structure. (f) Overview (50 × 50 nm^2^) and (g, h) close-up (15 × 15 nm^2^) STM images obtained after annealing to 653 K. **ZnTAP-20H** molecules form different size oligomers via intermolecular C–C coupling. (i) Overview STM image (50 × 33.2 nm^2^) of a sample with very high molecular coverage obtained after annealing to 653 K with molecules appearing as up/down bowls. (j) Simulated unoccupied state density maps of **ZnTAP-24H** molecules appearing as up/down bowls and **ZnTAP-22H** molecules with the Au adatom.

STM experiments performed on **ZnBAP** and **ZnTAP** molecules on the Au(111) substrate are summarized in Figures 2 and 3, respectively. Larger-scale overview images are shown in the Supporting Information.

Upon deposition onto a clean Au(111) substrate, **ZnBAP** molecules arrange into interwoven structures, as depicted in Figure 2a. In the lower left corner of the image, an island exhibiting low contrast is observed from the assembled impurities, most likely the leftover solvents from the synthesis. The close-up image shown in Figure 2b is overlaid with ball-and-stick molecular models obtained from force-field molecular modeling. Extended Hückel simulated unoccupied state density maps of such arranged molecules are shown in Figure 2c. The molecules in the middle of the interwoven structure form a linear chain through overlapping phenyl rings; the molecules on the outer edge assemble around them through the interaction of anthracenyl units.

The STM image shown in Figure 2d was obtained after annealing to 468 K, a temperature where loss of 8 H atoms occurs. **ZnBAP-8H** molecules were imaged as flat rectangles with bright protrusions at the middle of the long edge. Such a shape is consistent with the fusion of anthracenyl units to the porphyrin macrocycle, with phenyl rings appearing as protrusions. This is evident in the close-up image in Figure 2e that is superimposed with the molecular structures as well as in the simulated occupied state density maps shown in Figure 2f. Molecules assemble mostly onto the fcc facet of Au(111) along the herringbone reconstruction in the form of a chain. The assembly is promoted via interaction with the adjacent phenyl groups. Some molecules appear to be further dehydrogenated and resemble **ZnBAP-12H** (see below).

After annealing to 603 K and fusion of the phenyl groups to the macrocycle, **ZnBAP-12H** molecules were imaged completely flat (Figure 2g). Molecules are assembled randomly due to the absence of phenyl–phenyl interactions. The preference for the fcc facet of the herringbone reconstruction is still maintained. The molecules encircled in blue appear much brighter in the center due to Zn transmetalation with Au. Porphyrin metalation with Au adatoms has been shown to occur at similar temperatures.^54,59,60^ Some molecules appear to be missing one anthracenyl unit, most likely an impurity remaining from synthesis, products of fragmentation during sublimation, or side product of a surface reaction.

Both *anti-* and *syn-***ZnBAP-12H** species are observed (Figure 2g,h,i). On the account of three different images shown in Figure S1 in the Supporting Information, there is a prevalence for the formation of *anti-***ZnBAP-12H** with a yield of 70 ± 5% (Table S1 in the Supporting Information). This is well in accordance with previous work involving cyclodehydrogenation of metalated porphyrin molecules.^20^ Interestingly, for the molecules that are missing one anthracenyl unit (Zn-mono anthracenyl porphyrin, **ZnMAP**) the preference turns toward the *syn-* species with a yield of 59 ± 4% (Table S1 in the Supporting Information). There can be numerous reasons for such behavior reflected in the electronic structure of the **ZnMAP** molecule and the intermediate dehydrogenation products; however, detailed investigation thereof is beyond the scope of this manuscript.

Ordered islands of **ZnTAP** molecules are observed upon deposition on the Au(111) substrate (Figure 3a). Molecules adsorb ’parallel’ to the surface in a pinwheel configuration. A comparison of different adsorption geometries of **ZnTAP** is shown in Figure S2 and is accompanied by the simulated state density maps thereof. As evident from Figure S2, the pinwheel adsorption geometry provides the best building block for the observed molecular self-assembly. This is in contrast to the tetra-bromoanthracenyl-porphyrin row-like self-assembly on silver surfaces where edge-on adsorption geometry is observed.^61^ Self-assembly of **ZnTAP** is facilitated via the overlap of the anthracenyl units of neighboring molecules, similar to the case of **ZnBAP** molecules. The protruding anthracenyl units of four neighboring molecules are held together and appear bright in the image. Dark regions between them represent porphyrin macrocycles, as deducted from the edge of the molecular island. This is further corroborated via simulated unoccupied state density maps of **ZnTAP** molecules as arranged in the proposed model (Figure 3b).

Following annealing to 483 K and fusion of the anthracenyl units to the porphyrin macrocycle, **ZnTAP-16H** molecules are imaged as mostly flat with four pronounced protrusions (Figure 3c). The protrusions arise as a consequence of sterical overcrowding between the adjacent anthracenyl units and surface confinement. Six conformations are derived from the possibility to form *M-* or *P-* pentahelicene units in each of the four sites where anthracenyl units meet. Simulated occupied state density maps of those conformations are shown in Figure 3d. Labeling of the conformers is performed starting from the upper left corner, and the color code is used in Figure 3c as well as in Figure S3. Homochiral (*MMMM* and *PPPP*) conformers are the most abundant, while all of the other conformers could be identified in Figures 3c or S3. Their abundance is summarized in Table S2. Gray encircled molecules appear as if they are missing one anthracenyl unit, again an impurity from the synthesis or possibly a consequence of fragmentation during the sublimation or surface reaction.

Annealing to 613 K led to the formation of two additional C–C bonds between the adjacent anthracenyl units. The bonds form exclusively on the opposite sides of the **ZnTAP-20H** molecule (Figure 3e). The other two sides appear in the STM as a rather flat gap. Molecular modeling suggests that dehydrogenation might as well occur at these positions, but instead of the C–C bond formation, coordination with Au adatoms takes place. The inset within Figure 3e represents the simulated unoccupied state density map of such structure. A comparison of simulated images of several structures, namely, non-, partly, or fully dehydrogenated, as well as surface- and adatom-coordinated, is shown in Figure S4. The best match with the experimentally observed appearance is obtained in the case of dehydrogenation and Au adatom coordination (Figure S4e).

Further temperature increase and annealing to 653 K led to the polymerization of **ZnTAP-20H** molecules via intermolecular C–C coupling. Different sizes of oligomers were formed, as visible in Figure 3f. The coupling occurs next to the gap sides of the molecules (Figure 3g,h). This is in line with the previous assumption that dehydrogenation occurred at the two “gap” sides of the molecule, and the formed radicals were stabilized by adatom coordination. Similar behavior is observed in the case of open-shell [5]-rhombene molecule annealed on the Au(111) substrate.^62^

Interestingly, distinct situation occurs when a sample with very high molecular coverage, i.e., a full layer of **ZnTAP-16H**, was annealed to 653 K. Namely, molecules are here imaged either as circles with a darker region in the middle, or as bright circles with fading intensity from the middle outward (Figures 3i and S5). Such appearances are congruent with bowl-like molecular structures of **ZnTAP-24H** molecules adsorbed in bowl up/down configurations (Figure 3j). The up/down arrangement of adjacent bowls is expected to maximize the intermolecular π–π interaction and has been observed previously for C_38_ buckybowls on Cu(111).^63^ We have modeled the appearance of several different structures, namely, the adsorption of atomic and molecular hydrogen onto **ZnTAP-20H** molecules, transmetalation with Au or Zn demetalation. However, good agreement with the STM appearance is achieved only if indeed all four C–C bonds between the adjacent anthracenyl units in **ZnTAP-16H** are formed (Figure S6). Note that transmetalation of **ZnTAP-24H** molecules with Au cannot be completely excluded due to a similarity in the simulated state density maps in Figure S6. The formation of **ZnTAP-24H** molecules might at first be surprising, but there are examples in surface chemistry where coverage plays an important role in steering the surface chemistry.^41,64−66^ Moreover, already at the low coverage, there are indications that dehydrogenation occurs at all four sterically overcrowded centers of **ZnTAP-16H** molecules, in two of them, new C–C bonds were formed, while the other two were stabilized with surface adatoms. The stabilization of these molecular ends with adatoms would become difficult at very high coverages due to a limited availability as well as accessibility for Au adatoms. Additionally, lateral repulsion and compression of the molecular layer would push the molecules toward the formation of additional intramolecular C–C bonds. Notably, this does not preclude the formation of intermolecular C–C bonds but is probably a competing process. Occasionally, a bright protrusion at the molecular rim is observed. The protrusion could be interpreted as a formation of 3 out of 4 C–C bonds in the **ZnTAP-16H** molecule (**ZnTAP-22H** molecule with Au adatoms). The simulated state density maps thereof are shown in Figure 3j (right) and Figure S6g, corroborating such interpretation.

At intermediate molecular coverage, namely with 65% of **ZnTAP-16H** molecules (Figure S7a–c), annealing to 603 K already led to the intermolecular coupling, i.e., polymerization (Figure S7d–f). This temperature is lower than the temperature that was required to induce polymerization at the low coverage (∼17%) sample shown in Figure 3c,e,f. Such behavior is not surprising since higher coverage would increase the probability that two **ZnTAP-20H** molecules meet and form an intermolecular bond. A similar trend is observed in the ToF-SIMS temperature series of samples with higher molecular coverage, as shown in Figure S8a. Importantly, the presence of **ZnTAP-20H** signals is not observed here with an elevation in temperature. Instead, molecular dimers thereof appear upon annealing to 518 K (Figure S8b). Upon further annealing, specific signals disappear again due to the formation of larger oligomers and transmetalation.

### Transmetalation of Zn with Au

**Figure 4:**
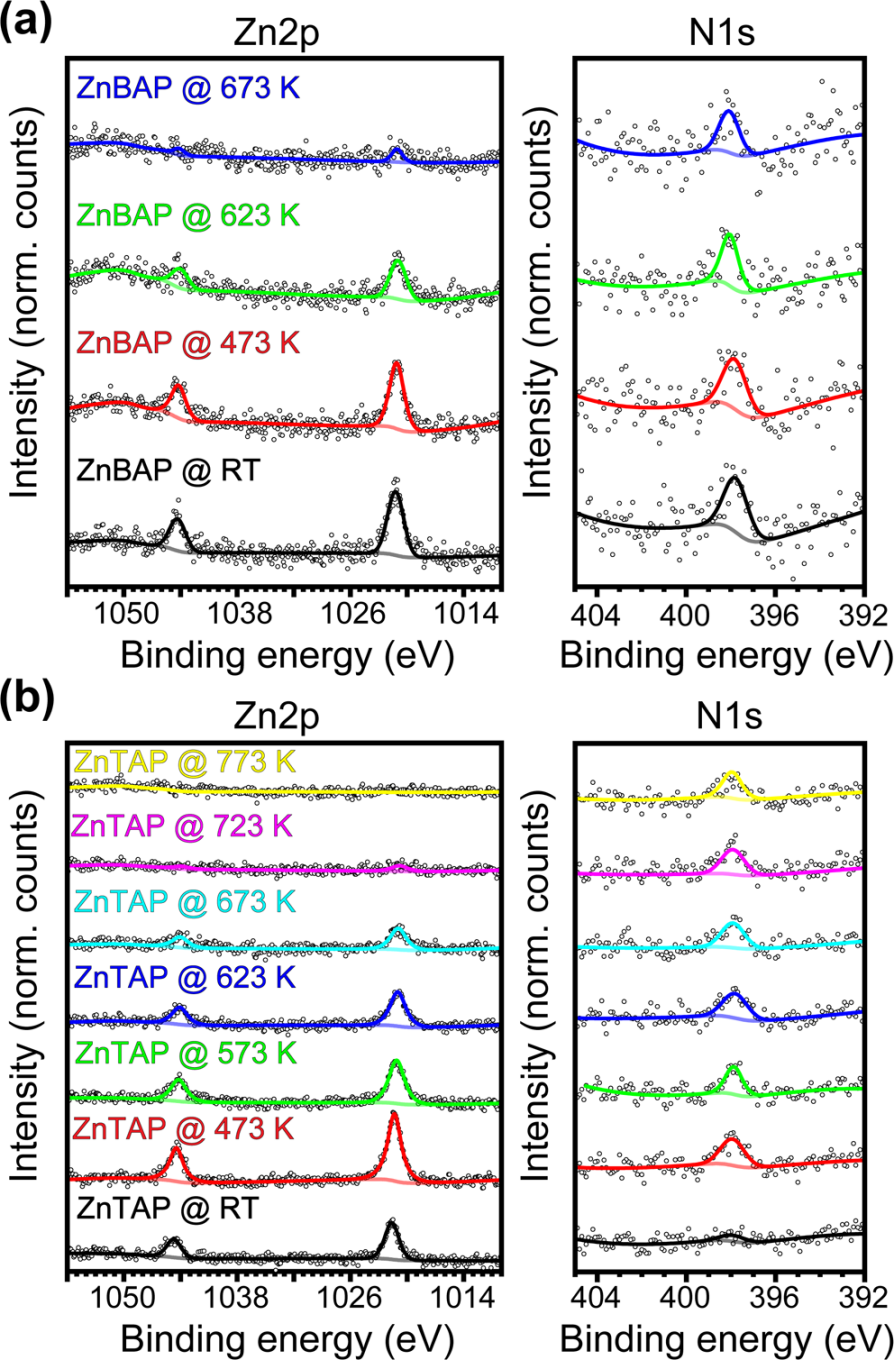
Temperature-dependent Zn 2p and N 1s XP spectra of **ZnBAP/ZnTAP** on Au(111). (a) Zn 2p and N 1s XP spectra obtained upon deposition and annealing of **ZnBAP** molecules on the Au(111) substrate. (b) Zn 2p and N 1s XP spectra obtained upon deposition and annealing of **ZnTAP** molecules on the Au(111) substrate. Significant reduction of all XPS signals was observed at the first annealing step due to molecular desorption. Additional molecular deposition was therefore performed while keeping the substrate at 473 K.

Species with very bright protrusion in the middle of the molecule, i.e., in the porphyrin macrocycle, were also observed in the STM experiments. These were ascribed to the Au-containing porphyrins due to transmetalation. In order to shed light on the transmetalation process, XPS was employed. Temperature-dependent Zn 2p and N 1s XP spectra of **ZnBAP** and **ZnTAP** molecules are shown in Figure 4a,b, respectively.

For both molecules, Zn 2p_3/2_ peaks appear at a binding energy of 1021.2 ± 0.2 eV. N 1s peaks appear at the binding energy of 397.9 ± 0.2 eV, exhibiting a single component. This is in very good agreement with XPS studies of other Zn-porphyrins adsorbed on Au(111).^67,68^ Upon annealing, Zn 2p signals gradually reduce and disappear at 773 K. During the Zn 2p signal reduction, almost no change is observed in the N 1s signals, neither in the intensity nor in the N 1s peak binding energy. This suggests that the Zn 2p signal decrease is due to demetalation and removal of Zn from the substrate via desorption or diffusion into the substrate and not to molecular desorption. Concurrently, metalation with Au occurs. The absence of higher-binding energy aminic N 1s feature at any annealing step suggests that the Zn demetalation and Au metalation occurred simultaneously, in a so-called transmetalation/atom-exchange process. Transmetalation of porphyrins and pyrphyrins on surfaces has been reported earlier,^69−71^ however, to the best of our knowledge not with Au. Note that significant reduction of all XPS signals was observed at the first annealing step of **ZnTAP** molecules due to molecular desorption; in order to circumvent that, additional molecular deposition was performed while keeping the substrate at 473 K. The Au transmetalation is also evident in **AuTAP, AuTAP-16H**, and **AuTAP-20H** ToF-SIMS signals shown in Figure S8c,d, in particular at elevated temperatures when the originating signals of **ZnTAP** vanish.

## Conclusions

In summary, an in-depth study of the on-surface synthesis of fused Zn bis- and tetraanthracen-9-yl porphyrins (**ZnBAP** and **ZnTAP**) by cyclodehydrogenation of the corresponding *meso*-substituted porphyrins on the Au(111) surface is provided herein. Starting from **ZnBAP** and **ZnTAP**, sequential dehydrogenation with temperature elevation leads to novel π-extended products beyond the ones obtained by wet synthesis. The products were identified in a combined STM, ToF-SIMS, and XPS study and are accompanied by molecular modeling. In the case of **ZnBAP** molecule, two cyclodehydrogenation steps are observed; in the first step, anthracenyl units fuse to the macrocycle (**ZnBAP-8H**). In the second step, phenyl groups fuse to the macrocycle in both *anti-* and *syn-*configurations (**ZnBAP-12H**). In the case of **ZnTAP** molecule, first the anthracenyl units fuse to the porphyrin macrocycle leading to the π-extended Zn porphyrin–helicene hybrid (**ZnTAP-16H**). The products of further annealing depend on the molecular coverage. At low coverage, two additional bonds are formed (**ZnTAP-20H**) and lead to molecular polymerization with temperature increase. At high coverage, three to four additional bonds form and lead to the formation of bowl-shaped porphyrin products (**ZnTAP-22H** and **ZnTAP-24H**). The overview of the observed surface products is summarized in Figure S9. In addition, the cyclodehydrogenation process is accompanied by the transmetalation of Zn with Au. The particular results demonstrate a facile strategy for the access to numerous anthracenyl-based π-extended porphyrin products that are of interest in the fields of molecular (photo/electro)catalysis, (opto)electronics, and spintronics via cyclodehydrogenation and transmetalation.

## Experimental Section

### Anthracenyl Porphyrin Synthesis

**ZnBAP** and **ZnTAP** molecules were synthesized according to Scheme S1, and the full details about the synthesis and product characterization are provided in the Supporting Information. In short, **ZnBAP** was synthesized in four steps from 5,15-diphenylporphyrin: Zn metal incorporation, iodination at the *meso* position accompanied by demetalation, attachment of anthracenyl units, and finally metalation with Zn.^48^**ZnTAP** was synthesized according to the method developed by Volz and Schäffer.^49^ Briefly, 9-bromoanthracene was treated with *n*-butyl lithium which reacted with pyrrole-2-carboxaldehyde. The carbinol intermediate was tetramerized to give tetra-anthracenyl porphyrin. Finally, it was metalated with Zn.

### Sample Preparation

All experiments were performed under ultrahigh vacuum (UHV) conditions, and samples were prepared in situ. The Au(111) single crystal was cleaned by repetitive Ar^+^ sputtering and annealing cycles, and its cleanliness was confirmed. **ZnBAP** and **ZnTAP** molecules were evaporated from a homemade evaporator held at 633 K. The sample annealing temperature was controlled via a K-type thermocouple directly spot-welded onto the crystal.

### ToF-SIMS Measurements

ToF-SIMS measurements were performed with a ToF-SIMS 5 instrument (IONTOF GmbH) on the in situ prepared samples. A 25 keV beam of Bi_3_^+^ primary ions was randomly rasterized over an area of 0.25 mm^2^, and secondary ions were collected using an extraction voltage of 3 kV. For each measurement step, 150 spectra of negative and positive secondary ions were acquired at the same spot, while a new spot was chosen for each new measurement. The total dose density at each single spot was kept below 10^12^ ions per cm^2^, which provided the static limit without detectable sample alteration. Mass calibration was performed using the Au_n_^+^ cluster signals of the surface. Note that in ToF-SIMS ionization, probability plays a paramount role in signal detection; this in turn makes it very difficult to quantify molecular coverage and is therefore referred to as higher and lower according to the difference in evaporation time. Simulated isotope mass distributions shown above the spectra were calculated using the enviPat web package.^72^

### STM Experiments

STM experiments were performed in constant current mode with electrochemically etched tungsten tips. Two different scanning tunneling microscopes were employed, namely a variable-temperature STM (Omicron Nanotechnology GmbH) operated at 50 K and a home-built low-temperature scanning tunneling microscope, operated at 7 K. Images were analyzed using the WSxM software^73^ and filtered by global plane subtraction. The parameters of STM image acquisition are provided in Table S3 in the Supporting Information.

### XPS Measurements

XPS measurements were performed using a PHOIBOS 100 electron analyzer (Specs GmbH) in normal emission with a nonmonochromatized Al-Kα X-ray source. The binding-energy scale was calibrated using the substrate Au 4f peak position and the Fermi level of the Au crystal. All spectra were normalized to the Au 4f_7/2_ signals, and background signals obtained on the clean Au(111) crystal were subtracted. Spectra were then fitted using Gaussian/Lorentzian convolution functions for components with simultaneous optimization of the Shirley spectral background by using Unifit 2013 software.

### Molecular Modeling

Molecular modeling was performed using HyperChem 8.0 software. Structure optimization of molecules adsorbed onto a 3-layered Au(111) slab was performed using molecular mechanics (Amber force field) with periodic boundary conditions. Au atoms on the surface were kept fixed during the optimization. Occupied and unoccupied state density maps were calculated using extended Hückel theory of the optimized structures.

## Supporting Information Available

Detailed description of **ZnBAP/ZnTAP** synthesis; overview images; and abundance of fused **ZnBAP/ZnTAP** molecules; modeled conformations of the native **ZnTAP** molecule; modeled structures for **ZnTAP-20H** and **ZnTAP-24H** molecules, as well as STM imaging parameters (PDF)
