# Supplementary material for: Planar and Curved π-Extended Porphyrins by On-Surface Cyclodehydrogenation
Source: J Am Chem Soc. 2024 Dec 4;146(50):34600–8. doi: 10.1021/jacs.4c12460 (PMC11664915; doi:10.1021/jacs.4c12460)
Supplement: Supplementary file 1 — ja4c12460_si_001.pdf [file ja4c12460_si_001.pdf]

Supporting Information

Planar and Curved π-Extended Porphyrins by On-Surface Cyclodehydrogenation

Miloš Baljozović,[†,*] Joffrey Pijeat,[‡] Stéphane Campidelli[‡,*] and Karl-Heinz Ernst[†,§,¶,*]

[†] Molecular Surface Science Group, Empa, 8600 Dübendorf, Switzerland

[‡] Université Paris-Saclay, CEA, CNRS, NIMBE, LICSEN, 91191 Gif-sur-Yvette, France

[§] Department of Chemistry, University of Zürich, 8057 Zürich, Switzerland

[¶] Nanosurf Laboratory, Institute of Physics, The Czech Academy of Sciences, 16200 Prague, Czech Republic

[*]Authors to whom correspondence should be addressed (milos.baljozovic@empa.ch, stephane.campidelli@cea.fr and karl-heinz.ernst@empa.ch)

Table of content:

- Details of the **ZnBAP** and **ZnTAP** synthesis
- NMR and MALDI-TOF spectra
- Figure S1: Fully fused **ZnBAP** molecules
- Table S1: Abundance of *anti-*/*syn-* fused **ZnBAP**/**ZnMAP** molecules
- Figure S2: Different conformations of native **ZnTAP** molecules
- Figure S3: Large scale images of **ZnTAP–16H** molecules
- Table S2: Abundance of different **ZnTAP–16H** molecules
- Figure S4: Comparison of native and Au coordinated **ZnTAP–20H** molecule
- Figure S5. Additional STM images of high coverage samples annealed to 653 K
- Figure S6: Comparison of possible structures for Figure 3i
- Figure S7. STM images upon annealing sequence of a sample with intermediate coverage (~65 %)
- Figure S8. Additional ToF-SIMS data on higher/lower coverage samples
- Figure S9. Overview of observed products
- Table S3: STM imaging parameters
- References

Details of the ZnBAP and ZnTAP synthesis

Reagents. 5,15-diphenylporphyrin (**1**) was purchased from PorphyChem. Chemicals were purchased from Sigma Aldrich or Fisher Scientific and were used as received. Solvents were purchased from SDS Carlo Erba and were used as received. For synthesis CH_2Cl_2 (CaH_2, N_2), toluene (K/benzophenone, N_2), THF (K/benzophenone, N_2), Et_2O (CaH_2, N_2) were distilled before use.

Techniques. NMR spectra were recorded with a Brüker Avance 400 (400 MHz) instrument with solvent used as internal reference. MS and HRMS MALDI-TOF spectra were recorded on a Brüker Autoflex maX or on a Brüker UltrafleXtreme. Absorption spectra were recorded in quartz cuvettes on a Perkin Elmer Lambda 900 UV-Vis-NIR spectrophotometer. Thin layer chromatography (TLC) was performed on silica gel 60 F254 (Merck) precoated aluminium sheets. Column chromatography was performed on Merck silica gel 60 (0.063-0.200 mm).

Synthesis. The synthesis of **ZnBAP** is depicted in Scheme S1a; it is synthesized in four steps from 5,15-diphenylporphyrin **1.**[1] The first step consists of the incorporation of zinc in the porphyrin to give **2** which is then iodinated in *meso* position with I_2 in the presence of silver hexafluorophosphate following a protocol derived from literature.[2] Iodinated porphyrin **3** reacts with anthracen-9-boronic acid in the presence of tris(dibenzylideneacetone)dipalladium (Pd_2dba_3) and 2-dicyclohexylphosphino-2′,6′-dimethoxybiphenyl (SPhos) to give the bis-anthracen-9-yl porphyrin **4** which is metalated with Zn using zinc-acetylacetonate ($Zn(acac)_2$) in toluene, leading to the Zn(II) bis-anthracen-9-yl porphyrin **5 (ZnBAP).**

ZnTAP is synthesized according to the method developed by Volz and Schäffer[3] (Scheme S1b). Briefly, 9-bromoanthracene is treated with n-butyl lithium (n-BuLi) and reacts with pyrrole-2-carboxaldehyde. The carbinol intermediate is tetramerized in propionic acid under reflux to give 5,10,15,20-tetra anthracen-9-yl porphyrin **6**. Finally, the **6** is metalated in the presence of zinc acetylacetonate to give Zn(II) tetra-anthracen-9-yl porphyrin **7 (ZnTAP)**. Both **ZnTAP** and **ZnBAP** as well as their precursors were purified using thin-layer chromatography (TLC) and characterized using several spectroscopic techniques. The yields of the respective products are indicating above them in the Scheme S1, while the reagents and conditions of the synthesis step are indicated in-between.

Scheme S1. Anthracenyl Porphyrin synthesis. (a) Synthesis of ZnBAP. (b) Synthesis of ZnTAP.

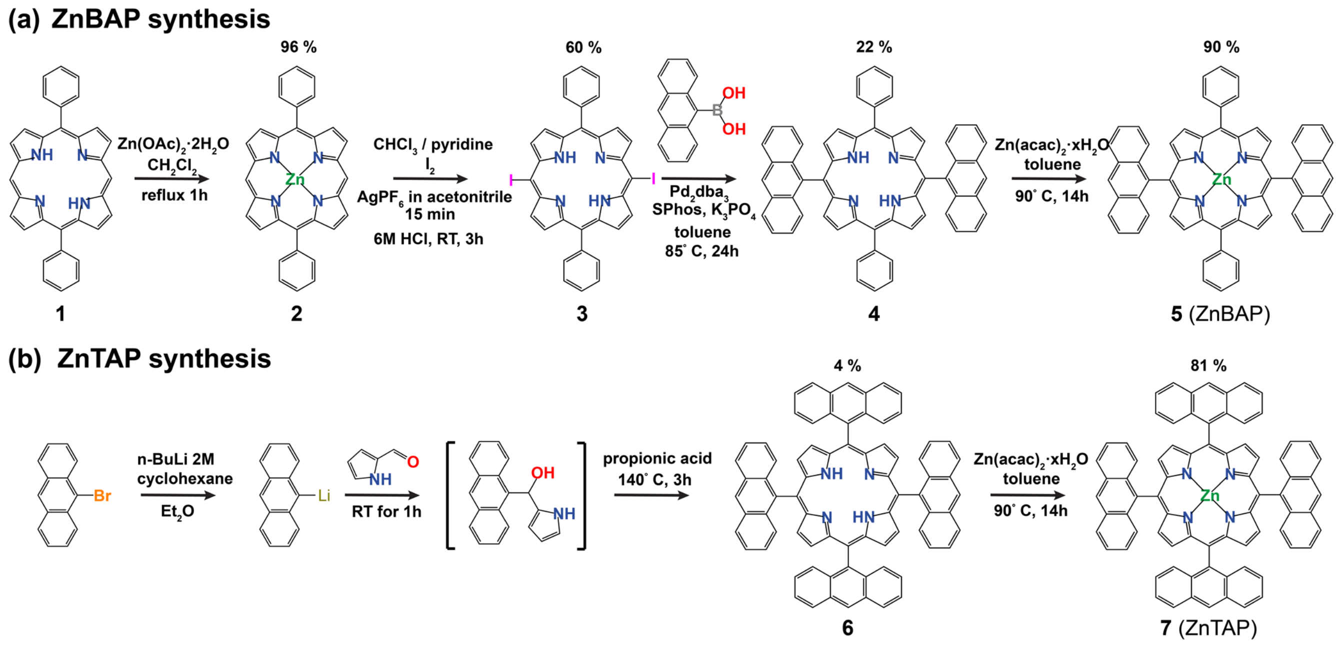

[5,15-diphenylporphyrinato]zinc(II) (2) synthesis. A solution of 5,15-diphenylporphyrin (**1**) (100 mg, 0.22 mmol) in CH_2Cl_2 (100 mL), $Zn(OAc)_2$ 2 H_2O (142 mg, 0.65 mmol) was refluxed for 1h. The reaction mixture was washed with water and the organic phase was dried over Na_2SO_4. After evaporation, **2** was precipitated in MeOH to afford violet-pink powder (109 mg,

96% yield). ^{1}**H NMR** (400 MHz, $CDCl_3$): δ 10.34 (s, 2H, meso), 9.45 (d, J = 4.4 Hz, 4H, β pyrroles), 9.15 (d, J = 4.4 Hz, 4H, β pyrroles), 8.32-8.22 (m, 4H, *m*-phenyls), 7.85-7.76 (m, 6H, *o*-phenyls + *p*-phenyls). **UV/Vis** (CH_2Cl_2): λ_{max} (log ε_{max}) = 412 (5.39), 541 (4.10). **MS (MALDI-TOF)** m/z $[M]^+$ calcd for $C_{32}H_{20}N_4Zn$: 524.10, found: 524.21.

5,15-diiodo-10,20-diphenylporphyrin (3) synthesis. Iodination of **2** was achieved by modification of literature procedure.[2] To a solution of **2** (50 mg, 0.19 mmol) and I_2 (194 mg, 0.76 mmol, 4 eq) in $CHCl_3$ (10 mL) and pyridine (300 μL) was added dropwise a solution of $AgPF_6$ (101 mg, 0.40 mmol, 2.1 eq) in MeCN (2 mL) and the mixture was stirred at room temperature for 15 min. The reaction was quenched by addition of water (10 mL) and the organic phase was extracted with $CHCl_3$ (3 x 30 mL), washed with water (3 x 30 mL) and dried over Na_2SO_4 before the solvent was removed under vacuum. The crude product was dissolved in $CHCl_3$ and a solution of HCl (6 M, 50 mL) was added to remove zinc. After 3h of stirring at RT, HCl was quenched by addition of $NaHCO_3$ and the combined organic phase were extracted with water (3 x 30 mL) and dried over Na_2SO_4 before the solvent was removed under vacuum. The crude product was purified by silica chromatography with $CHCl_3$/cyclohexane (1:2) with 1% Et_3N. After evaporation of solvent, **3** was obtained as a violet powder (41 mg, 60%). ^{1}**H NMR** (400 MHz, $CDCl_3$): δ 9.62 (d, J= 4.8 Hz, 4H, β pyrroles), 8.80 (d, J=4.8 Hz, 4H, β pyrroles), 8.18-8.13 (m, 4H, *m*-phenyls), 7.83-7.74 (m, 6H, *o*-phenyls + *p*-phenyls), -2.62 (s, 2H, H_{NH}). **UV/Vis** (CH_2Cl_2): λ_{max} (log ε_{max}) = 425 (5.48), 525 (4.14), 561 (4.09), 603 (3.65), 660 (3.77). **MS (MALDI-TOF)** *m/z* $[M+H]^+$ calcd for $C_{32}H_{20}I_2N_4$ 713.98, found: 713.94.

5,15-di(anthracen-9-yl)-10,20-diphenylporphyrin (4-BAP) synthesis. **3** (62 mg, 0.09 mmol, 1 eq), anthracen-9-boronic acid (77 mg, 0.34 mmol, 4 eq), Pd_2dba_3 (39.5 mg, 0.04 mmol, 0.5 eq) with SPhos (35.4 mg, 0.09 mmol, 1 eq) and K_3PO_4 (143.5 mg, 0.7 mmol, 8 eq) were dissolved in 30 mL of a degassed solution of toluene and water (1 mL), shielded from ambient light and stirred at 85°C for 24h. At the end of the reaction, the mixture was washed with water (3 x 40mL), the organic phase was dried over Na_2SO_4 and evaporated. The crude mixture was purified by two silica chromatography columns eluted with toluene/heptane (1:1), CH_2Cl_2/cyclohexane (1:1) and a SEC in distilled THF. After evaporation of solvents, **4** was obtained as a violet powder (16 mg, 22%). ^{1}**H NMR** (400 MHz, $C_2D_2Cl_4$): δ 8.98 (s, 2H, 10-anthracenyls), 8.71 (d, 4H, J = 4.8 Hz, β1), 8.34 (d, 4H, J =4.8 Hz, β2), 8.32-8.28 (m, 4H, 4,5-anthracenyls), 8.22-8.17 (m, 4H, *o*-phenyls), 7.72-7.67 (m, 6H, *m*-phenyls + *p*-phenyls), 7.54-7.48 (m, 4H, 3,6-anthracenyls), 7.19-7.14 (m, 4H, 1,8-anthracenyls), 7.10-7.05 (m, 4H, 2,7-anthracenyls), -2.25 (s, 2H, H_{NH}). ^{13}**C NMR** (100 MHz, $C_2D_2Cl_4$): δ 158.18, 141.35, 135.62, 135.06, 134.44, 130.68, 128.50, 128.24, 128.06, 127.67, 126.66, 125.72, 124.97, 120.10, 115.60, 99.36. **UV/Vis** (CH_2Cl_2): λ_{max} (log ε_{max}) = 422 (5.40), 517 (4.35), 550 (3.85), 591 (3.84), 647 (3.45). **MS (MALDI-TOF)** *m/z* $[M+H]^+$ calcd for $C_{60}H_{38}N_4$ 814.31, found: 814.47.

[5,15-di(anthracen-9-yl)-10,20-diphenylporphyrinato]zinc(II) (5-ZnBAP) synthesis. A solution of **4** (15.7 mg, 0.02 mmol) and $Zn(acac)_2$ hydrate (100 mg, 0.34 mmol – based on anhydrous material) was heated at 90°C in toluene overnight. The reaction mixture was washed with water and the organic phase was dried over Na_2SO_4 and evaporated. The crude was passed through silica pad (eluent CH_2Cl_2) to afford **ZnBAP** (15 mg, 90%). ^{1}**H NMR** (400 MHz, $C_2D_2Cl_4$): δ 8.97 (s, 2H, 10-anthracenyls), 8.80 (d, 4H, J = 4.8 Hz, β1), 8.38 (d, 4H, J =4.8 Hz, β2), 8.36-8.31 (m, 4H, 4,5-anthracenyls), 8.24-8.20 (m, 4H, *o*-phenyls), 7.72-7.65 (m, 6H, *m*-phenyls + *p*-phenyls), 7.53-7.47 (m, 4H, 3,6-anthracenyls), 7.15-7.09 (m, 4H, 1,8-anthracenyls), 7.08-7.01 (m, 4H, 2,7-anthracenyls). ^{13}**C NMR** (100 MHz, $C_2D_2Cl_4$ + a drop of CD_3OD): δ 151.00, 149.88, 142.56, 136.99, 135.10, 134.40, 132.13, 131.45, 130.65, 128.67, 127.54, 127.17, 126.32, 125.36, 124.83, 120.49, 115.93. **UV/Vis** (CH_2Cl_2): λ_{max} (log ϵ_{max}) = 255 (5.21), 422 (5.37), 516 nm (3.52), 552 (4.18). **MS (MALDI-TOF)** *m/z* $[M]^+$ calcd for $C_{60}H_{36}N_4Zn$ 876.22, found: 876.43.

5,10,15,20-tetra(anthracen-9-yl)porphyrin (6-TAP) synthesis. Porphyrin **6** was prepared by following the procedure established by Volz and Shäffer.[3] To a solution of 9-bromoanthracene (5.0 g, 19.46 mmol, 1 eq)) in dry diethyl ether (30 mL) at room temperature under Ar was added *n*-BuLi (10 mL, 20.0 mmol, 1.03 eq), the reaction mixture was stirred at room temperature for 30 min. Then, a solution of pyrrole-2-carboxaldehyde (925 mg, 9.7 mmol, 1 eq) in diethyl ether (10 mL) was added and the mixture was stirred for 1 h. The deep red mixture was poured into a cold and saturated solution of NH_4Cl (60 mL). The organic phase was separated, washed with water (3 x 30 mL) and dried over Na_2SO_4 before the solvent was removed under vacuum. The intermediary crude product was directly introduced into a boiling solution of propionic acid (50 mL), stirred for 3h at 140°C and allowed to cool overnight. 100 mL of MeOH was added and the black mixture was filtered over paper filter and washed with MeOH until the elution of a clear solution. The collected precipitate was poured into MeOH (500 mL), sonicated for 2 min and filtered again. The precipitate was purified on a large silica pad eluted with CH_2Cl_2 to collect the very first red-brown fraction.

Additional purification by silica chromatography with $CHCl_3$/cyclohexane (1:2) afford **6** as violet crystals (95 mg, 4% with respect to pyrrole-2-carboxaldehyde). *Note: the synthesis of porphyrin in propionic acid and air is known to form chlorin byproducts; if needed the porphyrin containing a small amount of chlorin is oxidized by DDQ in toluene following literature procedure.*[4] **^{1}H NMR** (400 MHz, $C_2D_2Cl_4$): δ 8.89 (s, 4H, anthracenyl), 8.34-8.18 (m, 8H, anthracenyl), 8.12 (s, 8H, β-pyrroles), 7.51-7.40 (m, 8H, anthracenyl), 7.32-7.24 (m, 8H, anthracenyl), 7.15-7.02 (m, 8H, anthracenyl), -1.75 (s, 2H, H_{NH}). *Note that because of the limited solubility of **6**, we only obtained a partial ^{13}C NMR spectrum*; **^{13}C NMR** (100 MHz, $C_2D_2Cl_4$): δ 135.00, 130.57, 128.54, 128.16, 128.01, 125.70, 124.89, 120.18, 115.94, 99.36, 79.76, 79.48, 79.20. **UV/Vis** (CH_2Cl_2): λ_{max} (log ϵ_{max}) = 255 (5.54), 258 (5.45), 354 (4.49), 375 (4.57), 426 (5.22), 519 (4.32), 548 (3.91), 592 (3.89), 657 nm (3.76). **MS (MALDI - TOF)** *m/z* $[M]^+$ calcd for $C_{76}H_{46}N_4$ 1014.54, found: 1014.37.

[5,10,15,20-tetra(anthracen-9-yl)porphyrinato]zinc(II) (7-ZnTAP) synthesis. A solution of **6** (30 mg, 0.03 mmol) and $Zn(acac)_2$ hydrate (50 mg, 0.19 mmol – based on anhydrous material) was heated at 90°C in toluene overnight. The reaction mixture was washed with water and the organic phase was dried over Na_2SO_4 and evaporated. The reaction mixture was washed with water and the organic phase was dried over Na_2SO_4. After evaporation, crude was precipitated in MeOH to afford violet-pink powder (109 mg, 96% yield). The crude was passed through silica pad (eluent CH_2Cl_2) to afford **ZnTAP** (26 mg, 81%). **^{1}H NMR** (400 MHz, $C_2D_2Cl_4$): δ 8.87 (s, 4H, 10-anthracenyls), 8.28-8.23 (m, 8H, 4,5-anthracenyls), 8.17 (s, 8H, β-pyrroles), 7.48-7.41 (m, 8H, 3,6-anthracenyl H), 7.26-7.20 (m, 8H, 1,8-anthracenyls), 7.02-7.02 (m, 8H, 2,7-anthracenyls). **^{13}C NMR** (100 MHz, $C_2D_2Cl_4$ + a drop of Pyridine-d5): δ 152.50, 133.25, 131.91, 129.94, 129.51, 128.85, 126.73, 126.06, 121.48, 117.36, 100.82, 81.19, 80.91, 80.63. **UV/Vis** (CH_2Cl_2): (λ_{max} (log ϵ_{max}) = 248 (5.14), 352 (3.95), 429 (5.00), 516 nm (3.52), 552 (3.98). **MS (MALDI-TOF)** *m/z* $[M]^+$ calcd for $C_{76}H_{44}N_4Zn$ 1076.29, found: 1076.42.

NMR (400 MHz) and MALDI-TOF spectra

^{1}H and MALDI-TOF spectra of [5,15-diphenylporphyrinato]zinc(II)

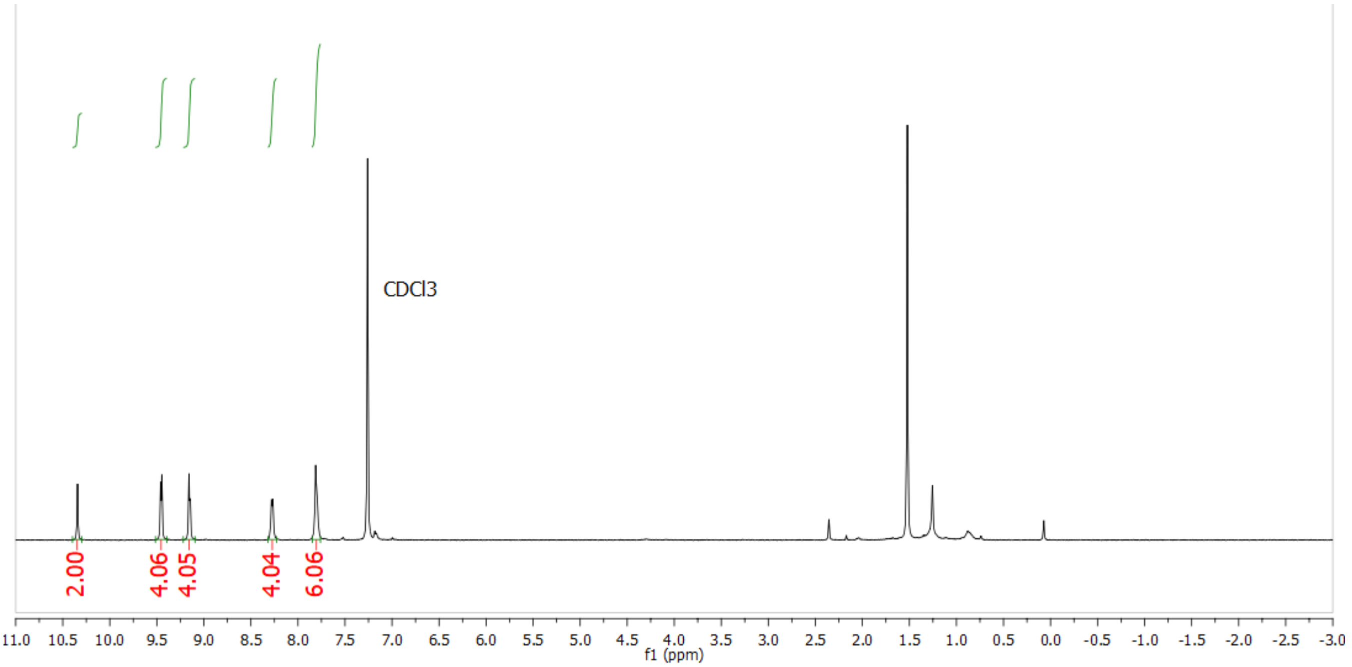

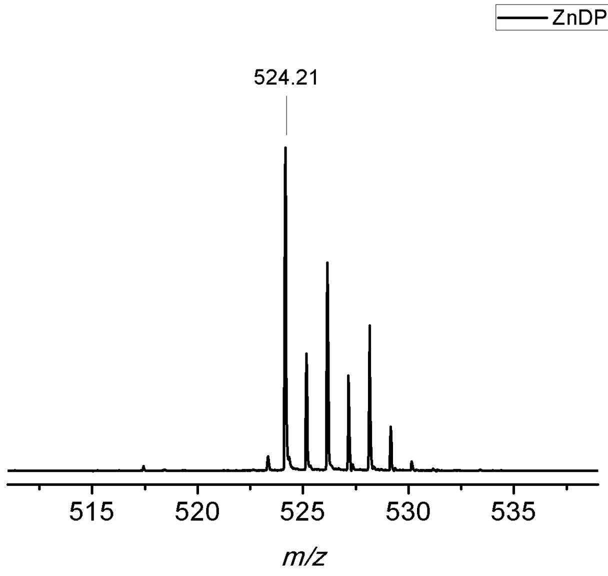

^{1}H and MALDI-TOF spectra of 5,15-diiodo-10,20-diphenylporphyrin (3)

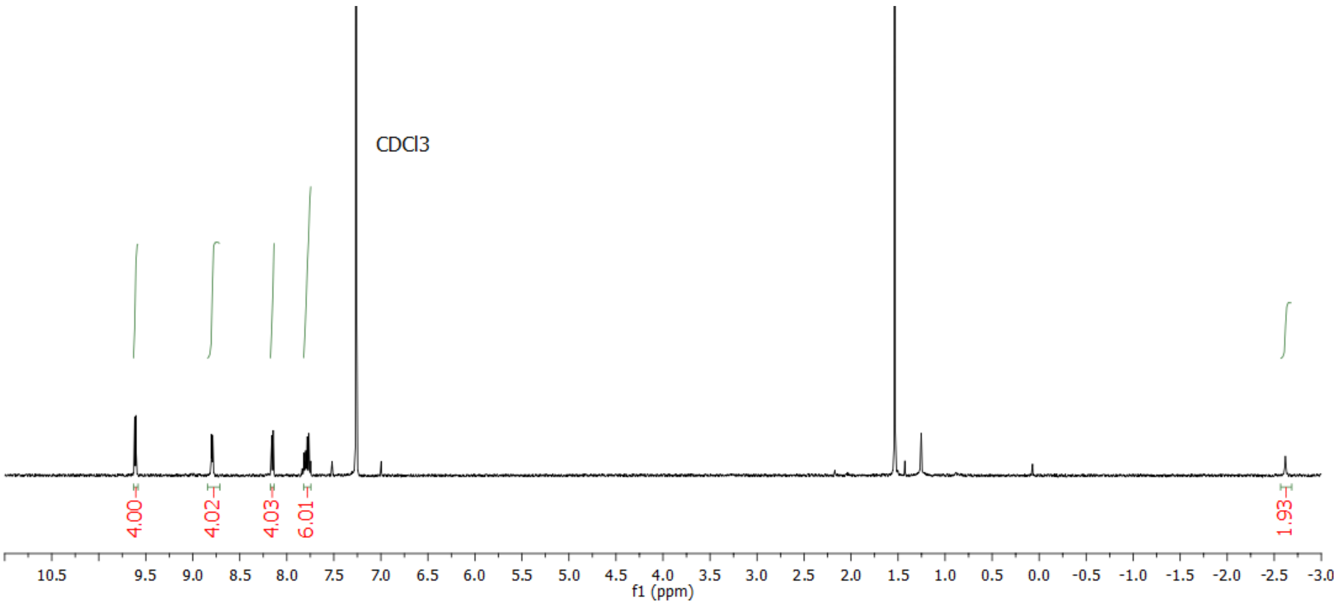

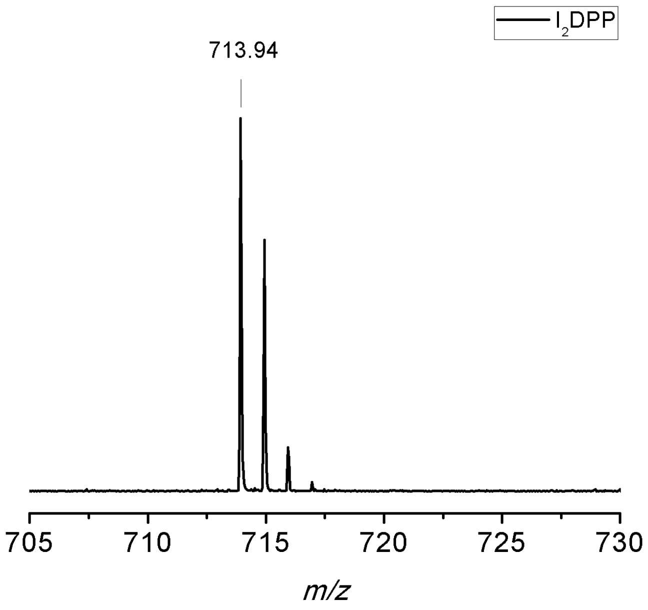

^{1}H, ^{13}C and MALDI-TOF spectra of 5,15-di(anthracen-9-yl)-10,20-diphenylporphyrin (4-BAP)

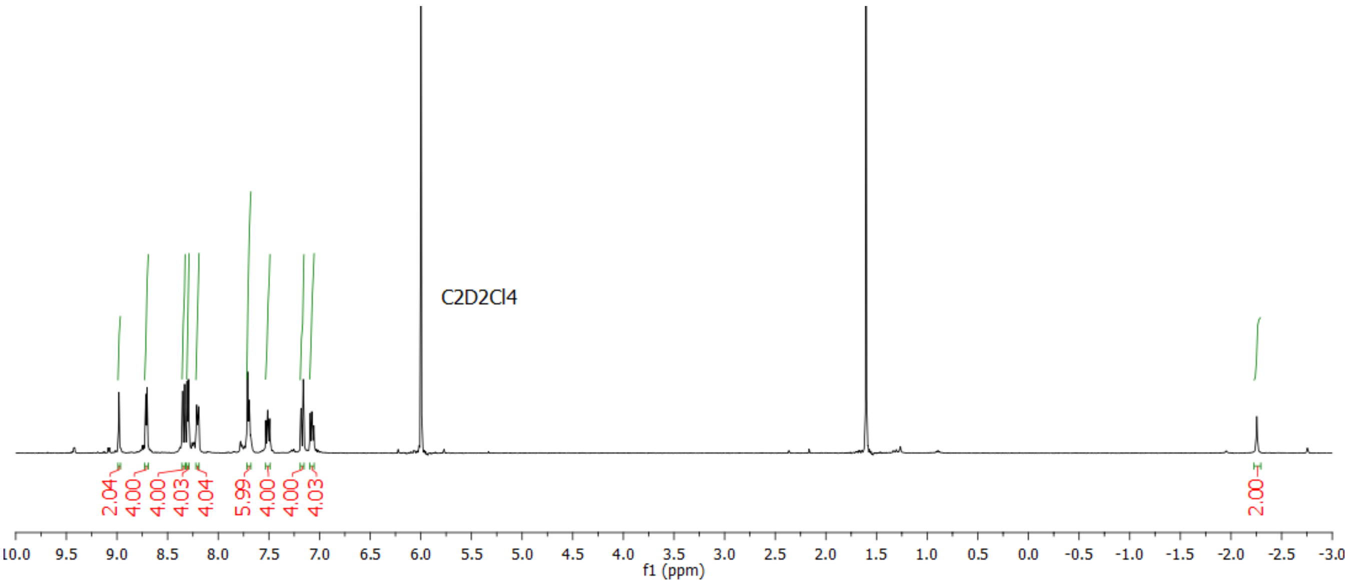

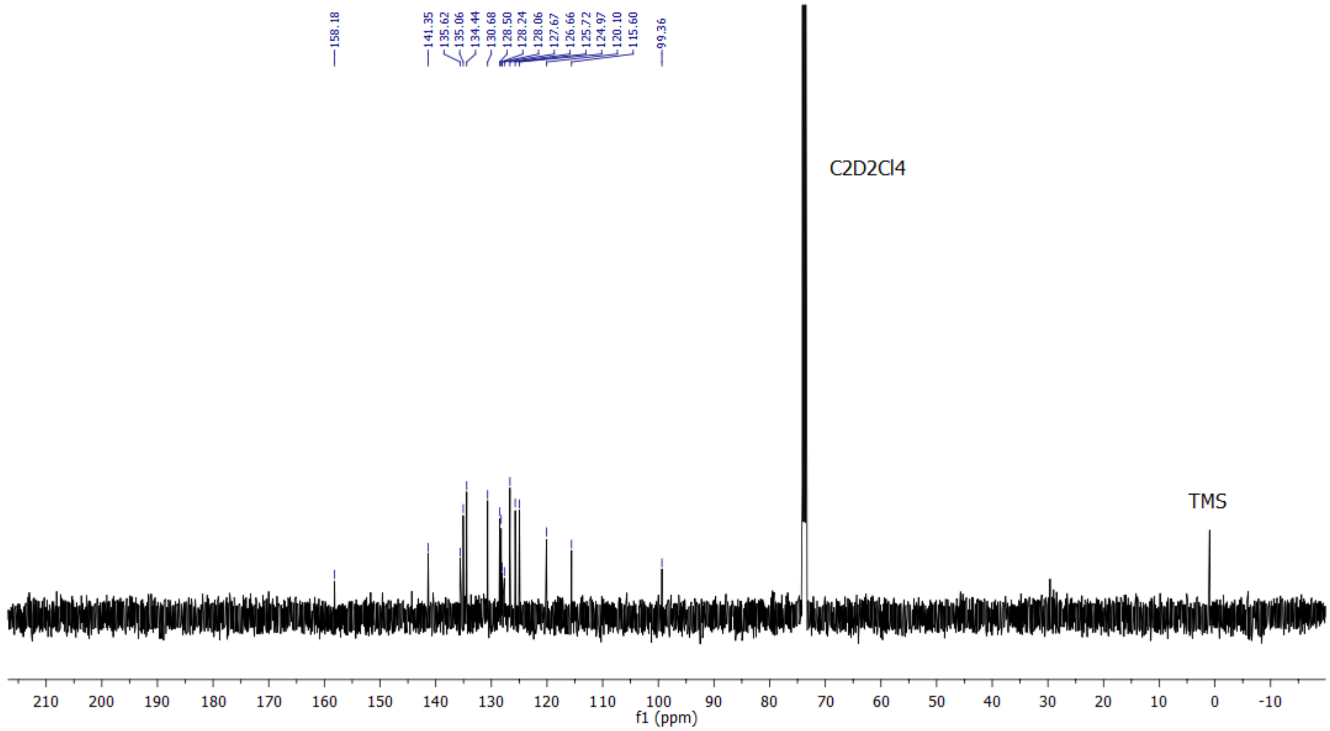

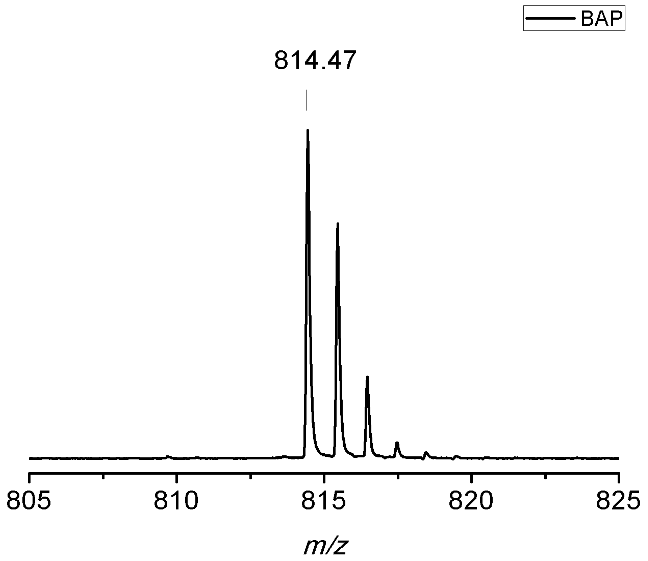
BAP
814.47
805
810
815
820
825
m/z

^{1}H, ^{13}C and MALDI-TOF spectra of [5,15-di(anthracen-9-yl)-10,20-diphenylporphyrinato]zinc(II) (5-ZnBAP)

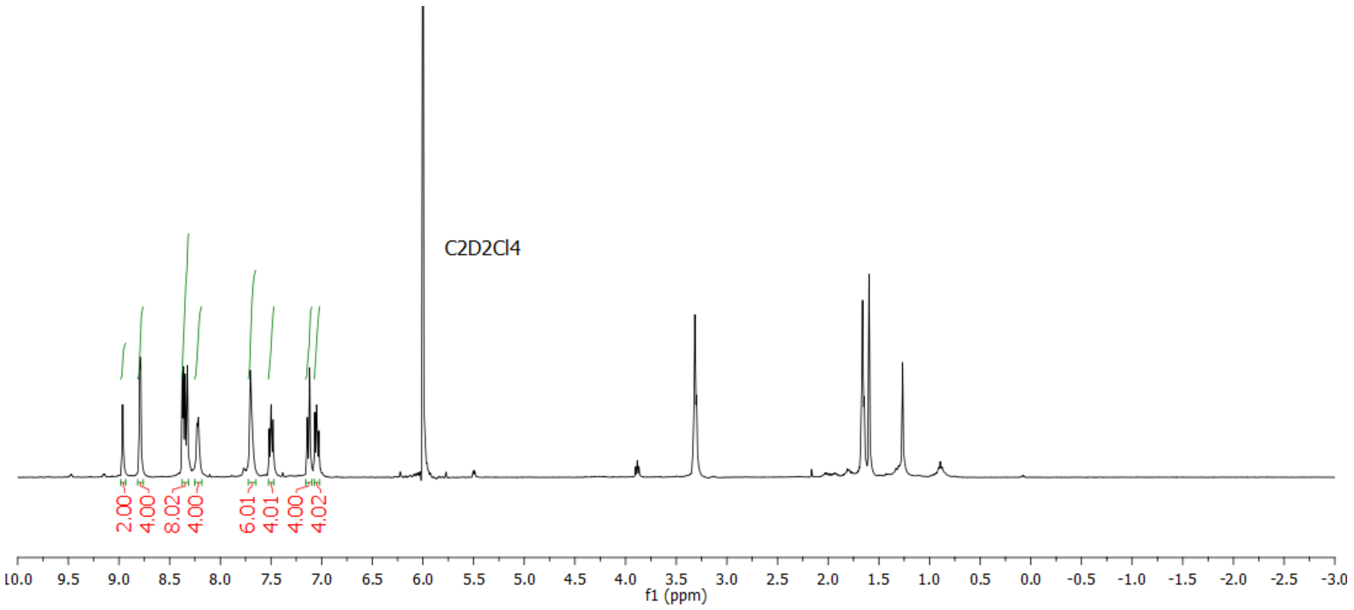

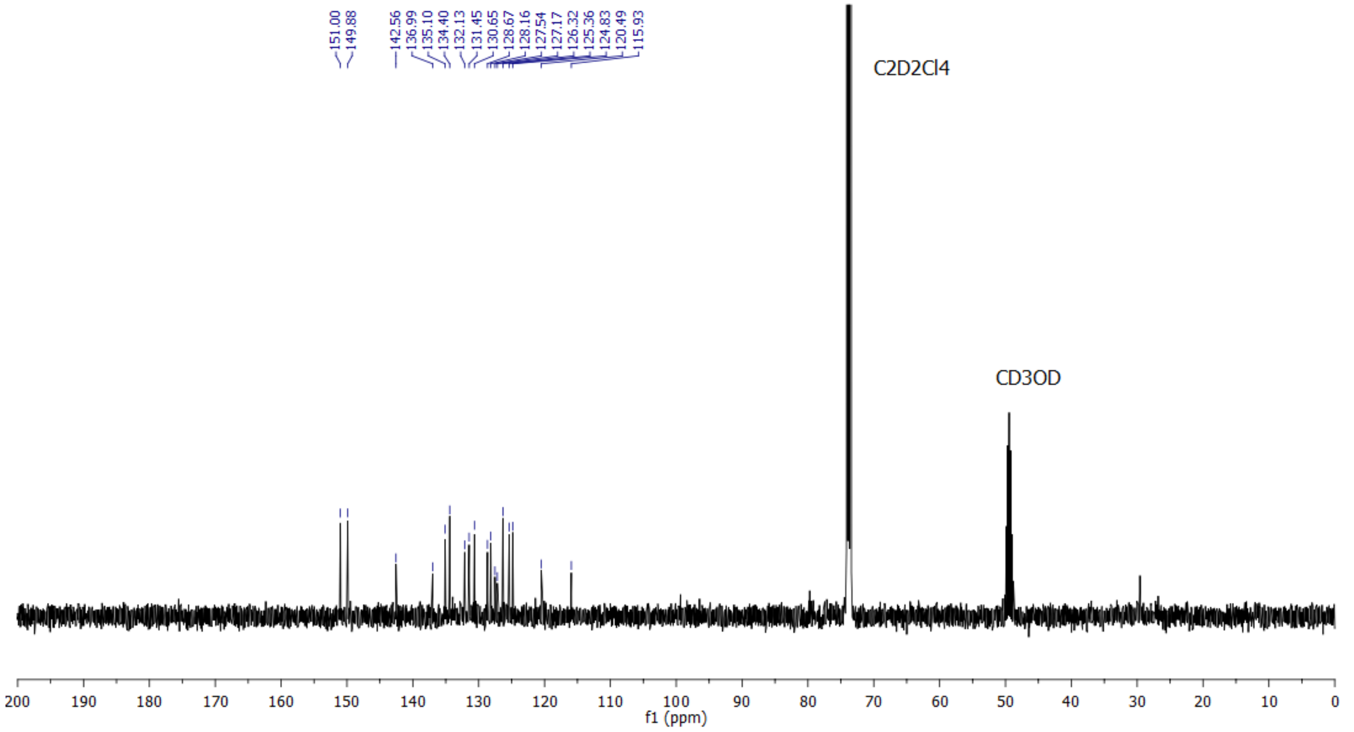

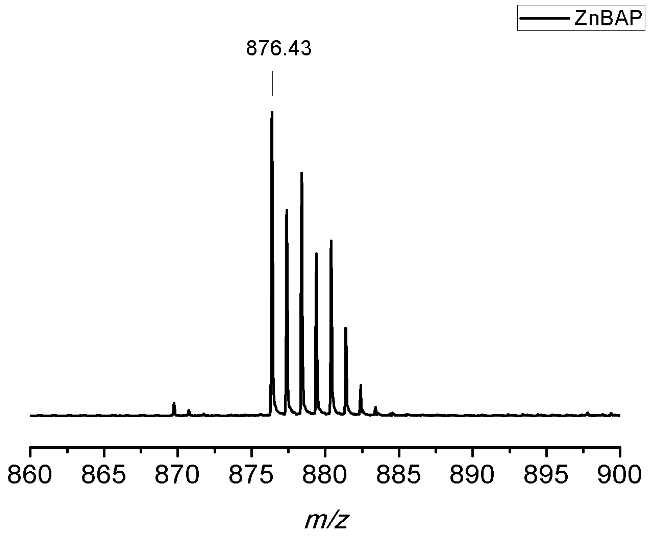
ZnBAP
876.43
860
865
870
875
880
885
890
895
900
m/z

^{1}H, ^{13}C and MALDI-TOF spectra of 5,10,15,20-tetra(anthracen-9-yl)porphyrin (TAP-6)

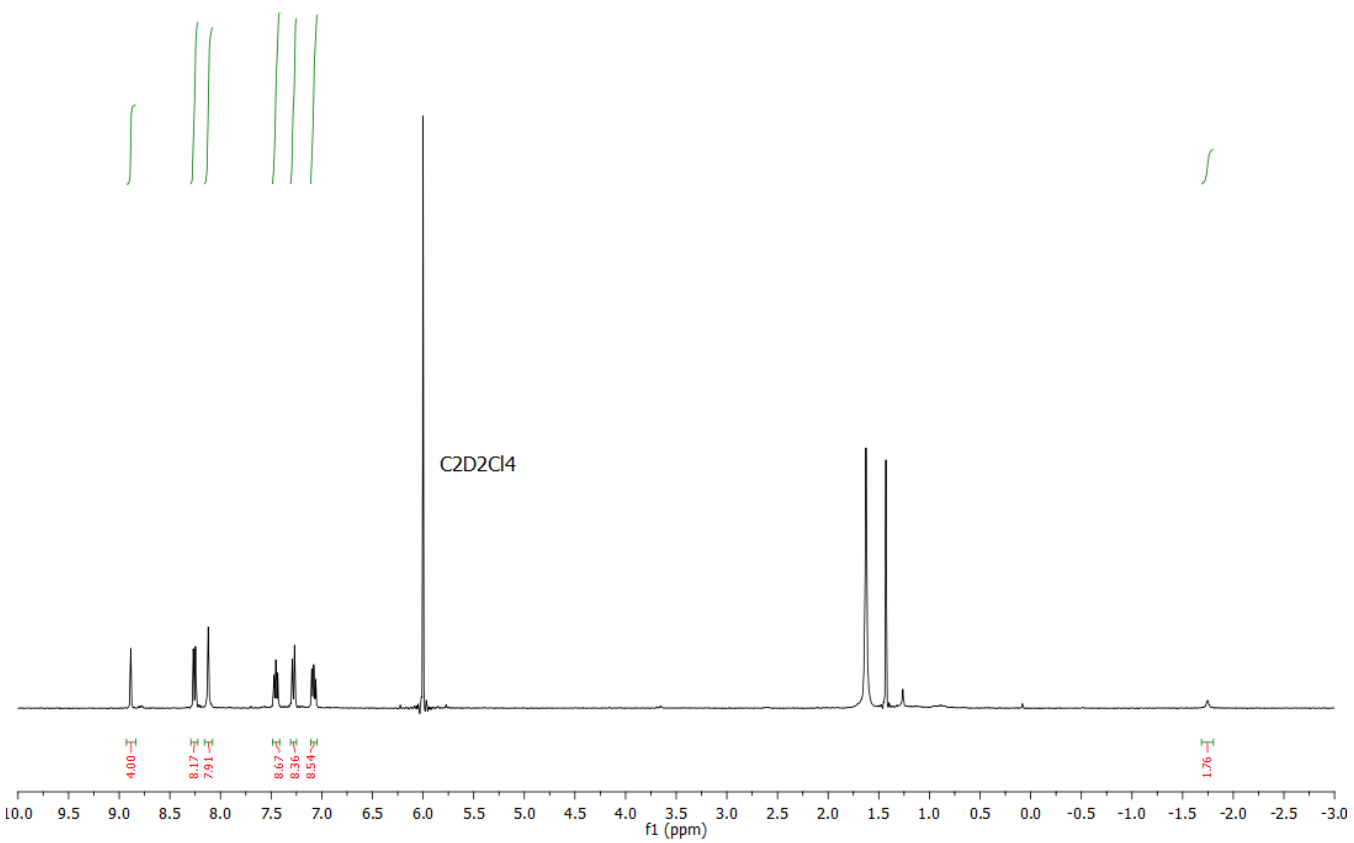

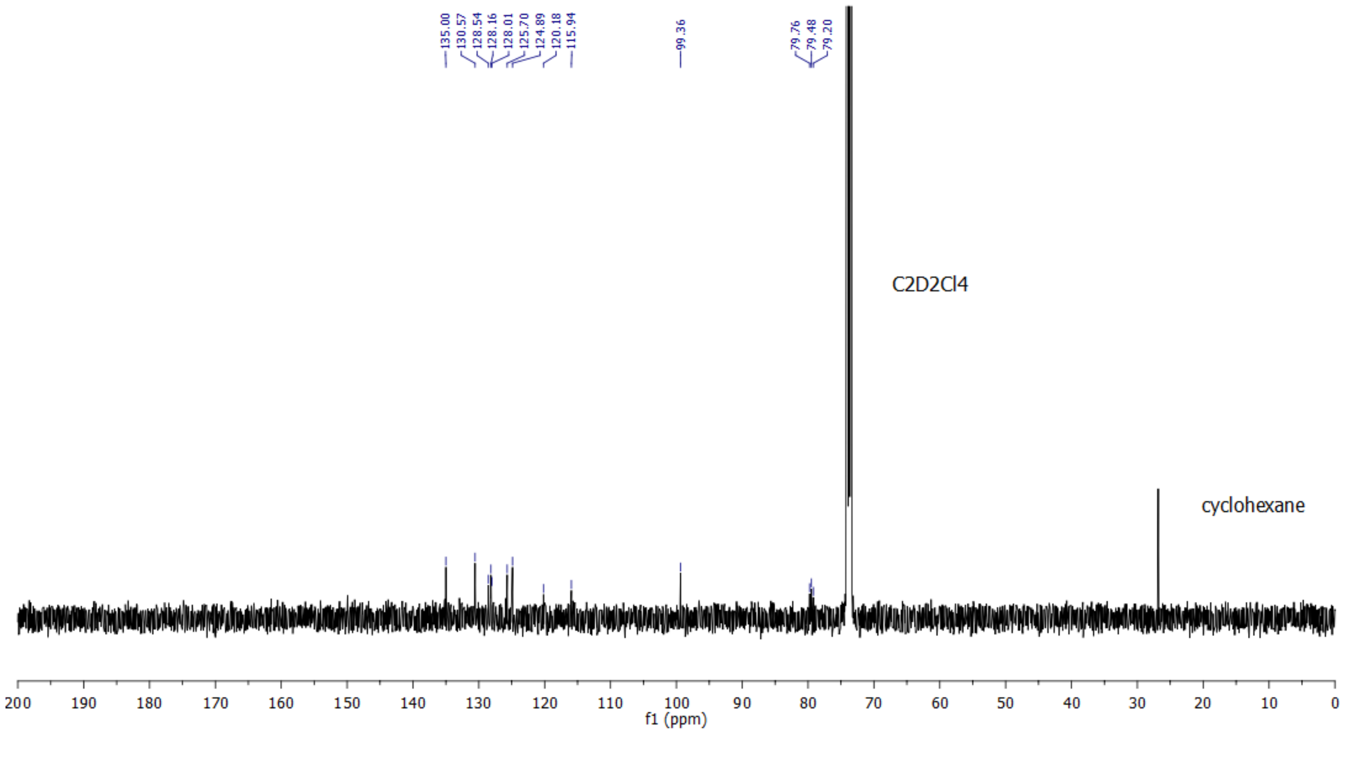

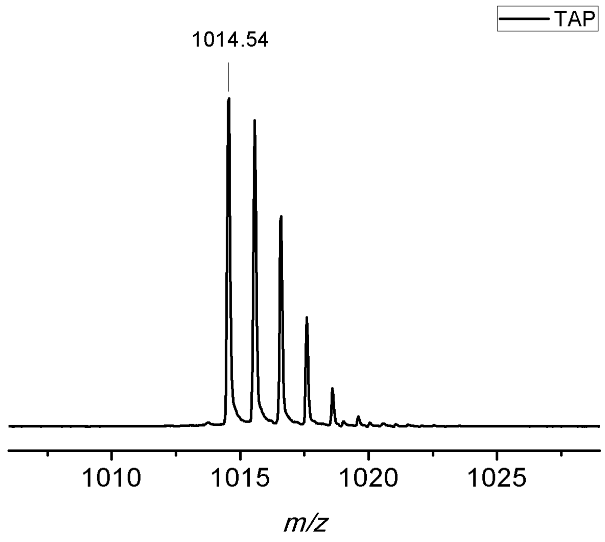
TAP
1014.54
1010
1015
1020
1025
m/z

^{1}H, ^{13}C and MALDI-TOF spectra of [5,10,15,20-tetra(anthracen-9-yl)porphyrinato]zinc(II) (ZnTAP)

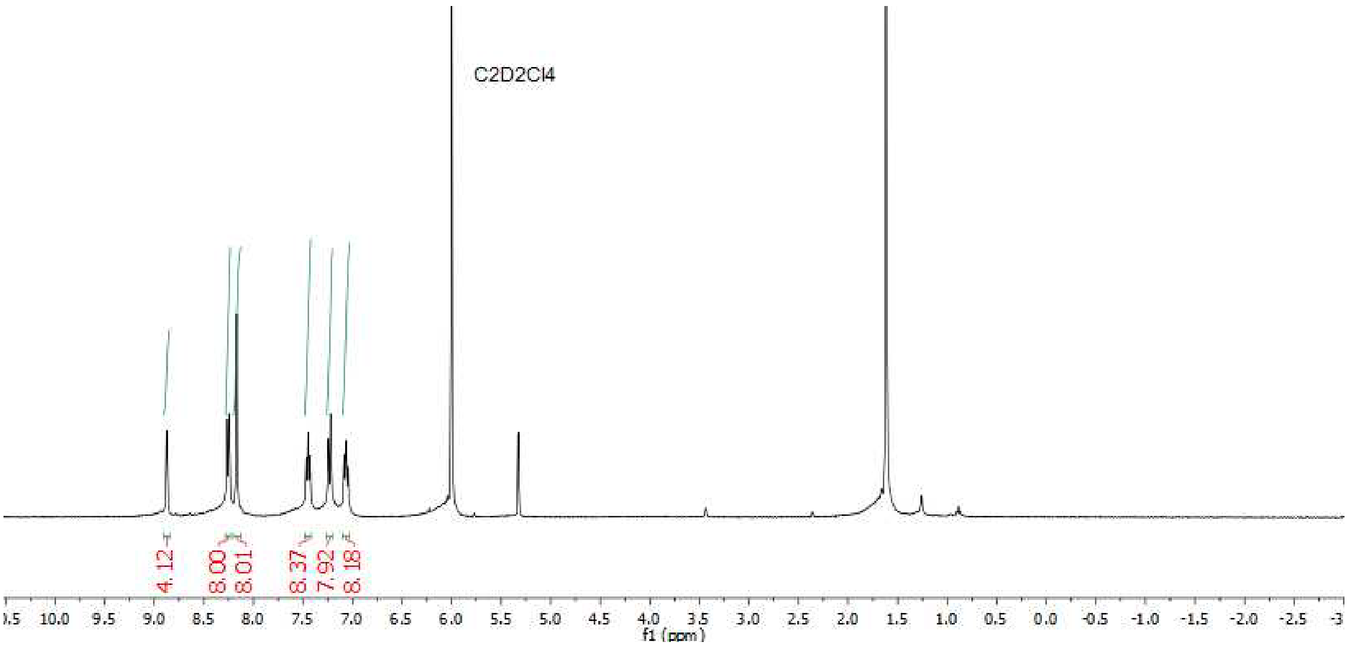

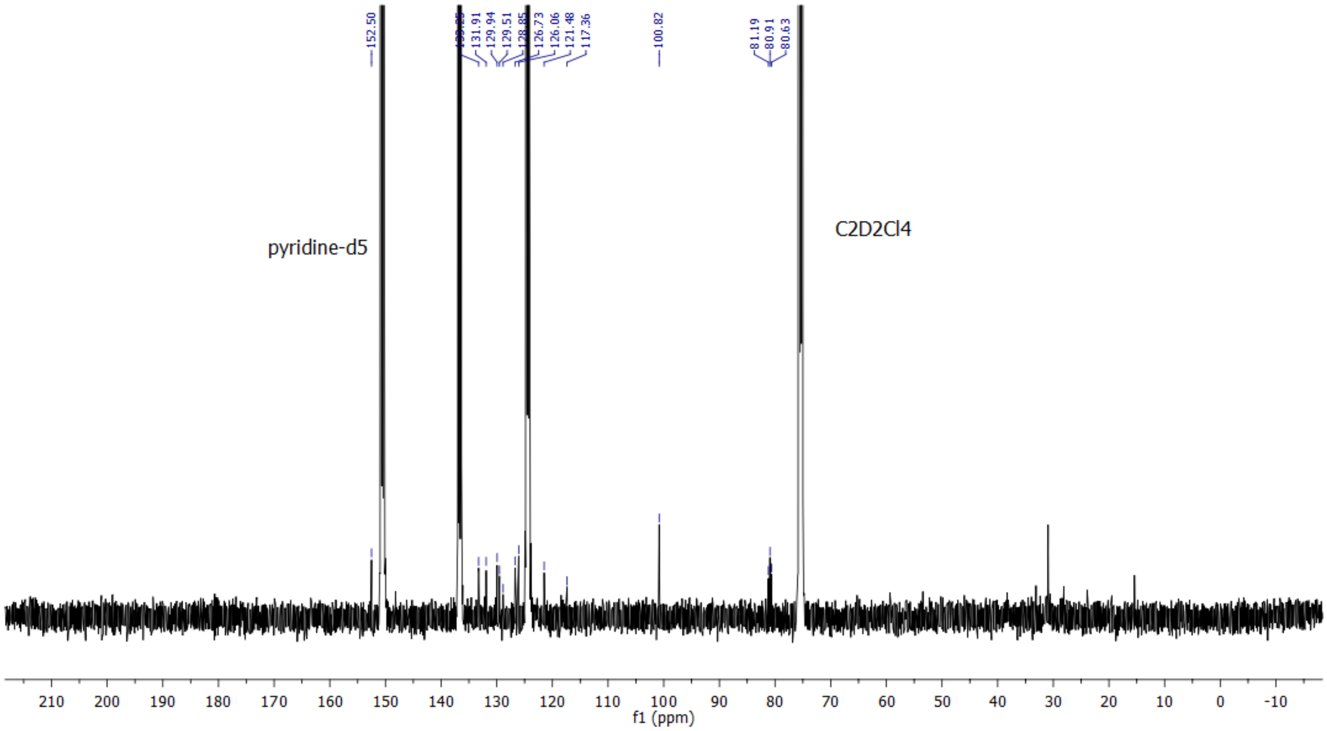

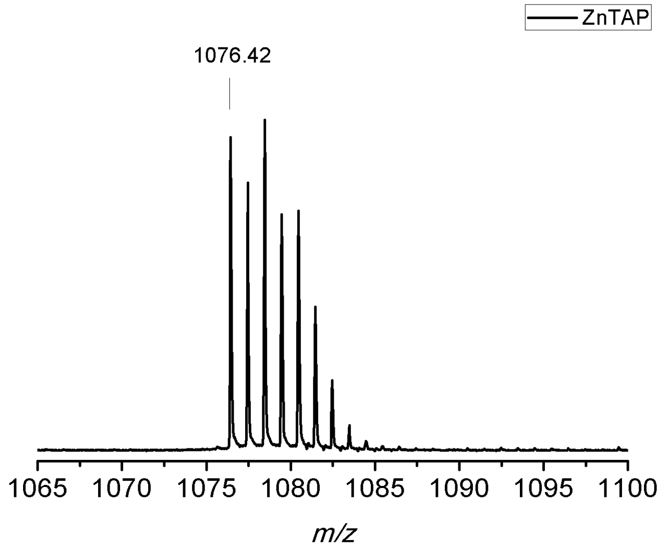
ZnTAP
1076.42
1065
1070
1075
1080
1085
1090
1095
1100
m/z

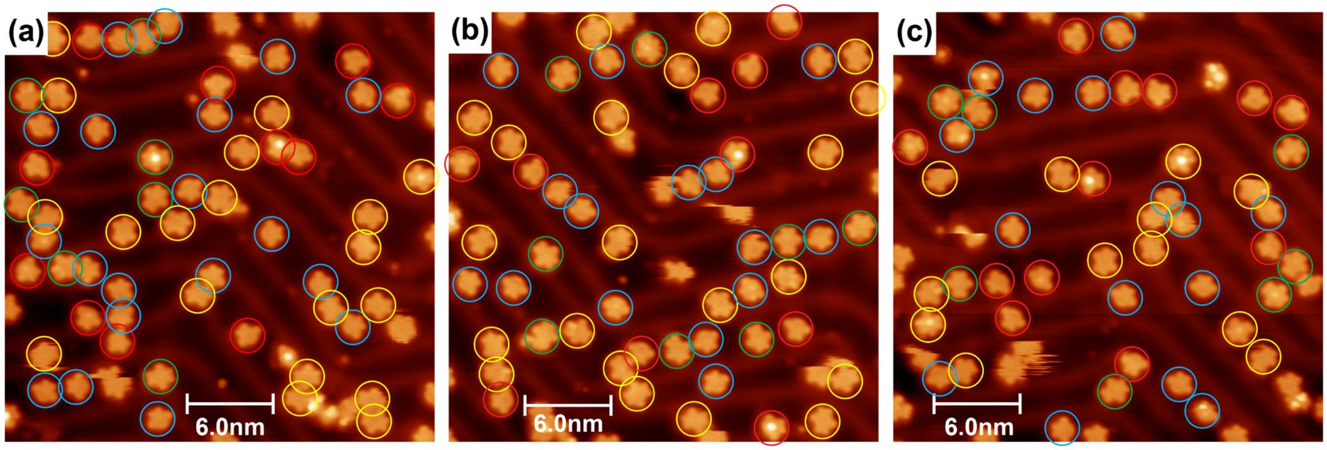

Figure S1. STM images of fully fused ZnBAP/ZnMAP molecules encircled in different colors depending on the structure of the fusion product. Yellow (*anti*- fused **ZnBAP**), green (*syn*- fused **ZnBAP**), red (*anti*- fused **ZnMAP**) and blue (*syn*- fused **ZnMAP**)

Table S1. Abundance of *anti-/syn-* fused ZnBAP/ZnMAP molecules

	Number of molecules			
**Figure**	*anti*- fused **ZnBAP**	*syn*- fused **ZnBAP**	*anti*- fused **ZnMAP**	*syn*- fused **ZnMAP**
Figure S1a	19	7	11	19
Figure S1b	21	8	11	15
Figure S1c	12	7	12	15
**Total**	**52**	**22**	**34**	**49**
**Percentage [%]**	**70 ± 5**	**30 ± 5**	**41 ± 4**	**59 ± 4**

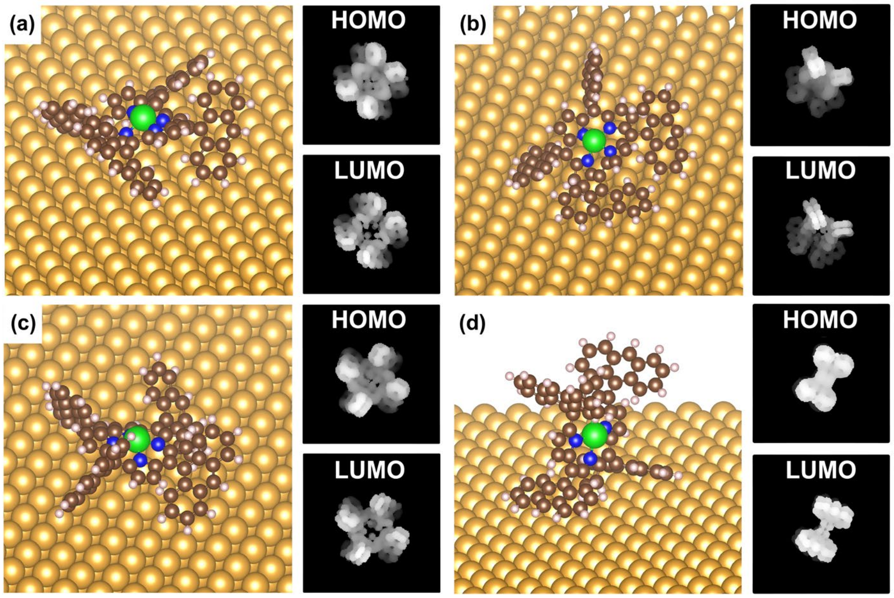

Figure S2. Comparison of different possible conformations of the native ZnTAP molecule together with simulated occupied and unoccupied density states maps thereof. (a) 'saddle shape' conformation with pairs of anthracenyl tips pointing in the same direction. (b) 'side' conformation with two anthracenyl units onto the substrate and the other two touching the substrate. (c) 'pinwheel' conformation with anthracenyl units tilted and pointing in the same direction in a clockwise or anticlockwise manner. (d) 'edge-on' conformation with two anthracenyl units in contact with the substrate and the other two hanging above.

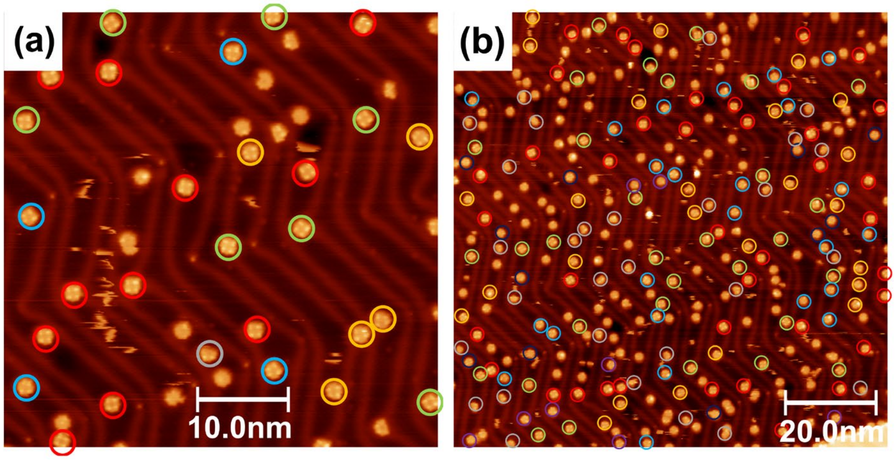

Figure S3. Additional STM images of ZnTAP–16H molecules encircled in different colors depending on the structure of the fusion product. The color code is assigned according to the Figure 3d.

Table S2. Abundance of different ZnTAP–16H molecules

	Number of molecules					
**Figure**	*MMMM*	*MMMP*	*MMPP*	*MPMP*	*MPPP*	*PPPP*	*unidentified*
Figure 3c	9	3	0	1	3	11	16
Figure S3a	7	3	1	0	5	11	14
Figure S3b	34	23	8	11	26	41	144

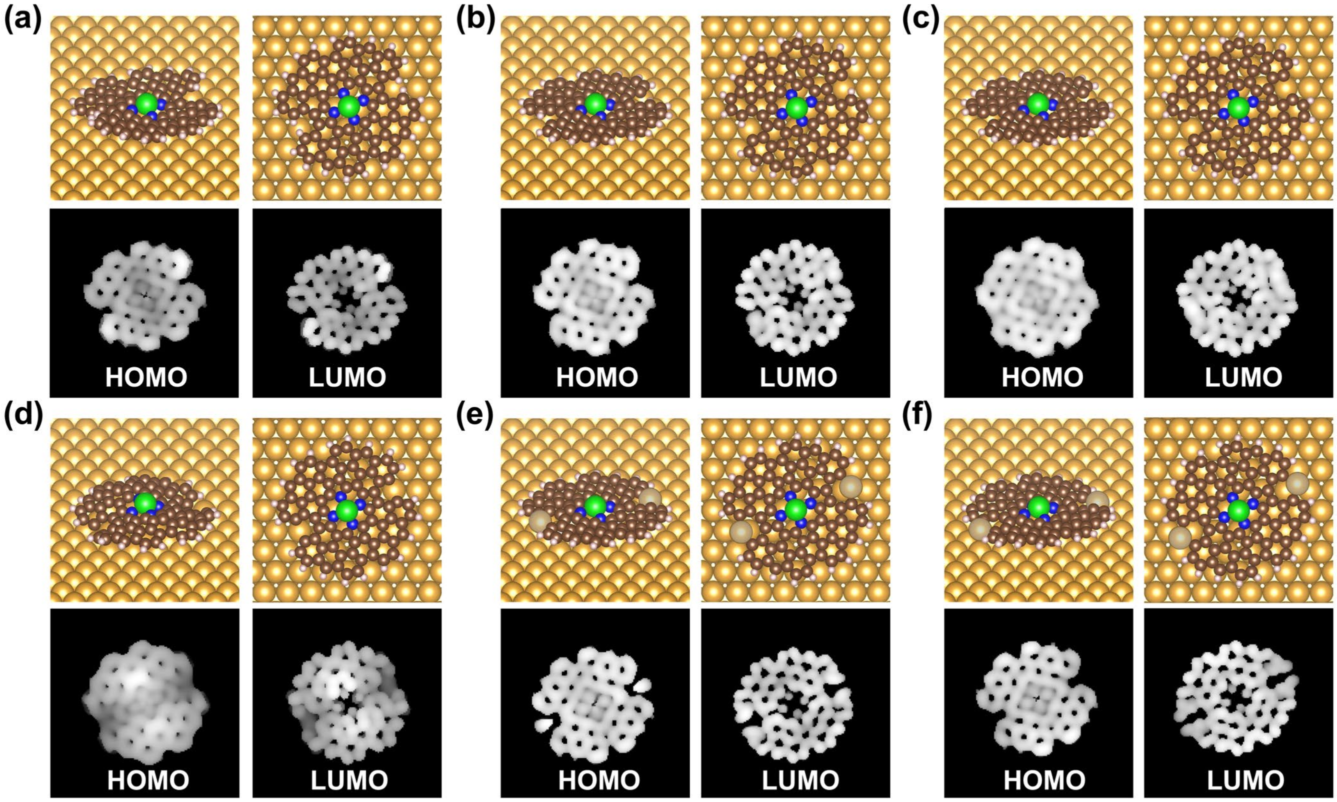

Figure S4. Comparison of optimized structures and occupied/unoccupied states density maps of several ZnTAP–20H derived models in an attempt to match experimental appearance from Figure 3e. (a) pristine **ZnTAP–20H**. (b) **ZnTAP–20H** with additional 2 hydrogen atoms removed at the 'gap' ends, one at each end. (c) **ZnTAP–20H** with additional 4 hydrogen atoms removed at the 'gap' ends, two at each end. (d) **ZnTAP–20H** with additional 4 hydrogen atoms removed at the 'gap' ends, two at each end and coordinated to the Au atom in the surface. (e) **ZnTAP–20H** with additional 4 hydrogen atoms removed at the 'gap' ends, two at each end and coordinated to the Au atom in the molecular plane. (f) **ZnTAP–20H** with additional 4 hydrogen atoms removed at the 'gap' ends, two at each end and coordinated to the Au atom sitting at the surface. The best agreement with the experimental appearance (Figure 3e) shows the structure in (e) with Au atoms in the molecular plane

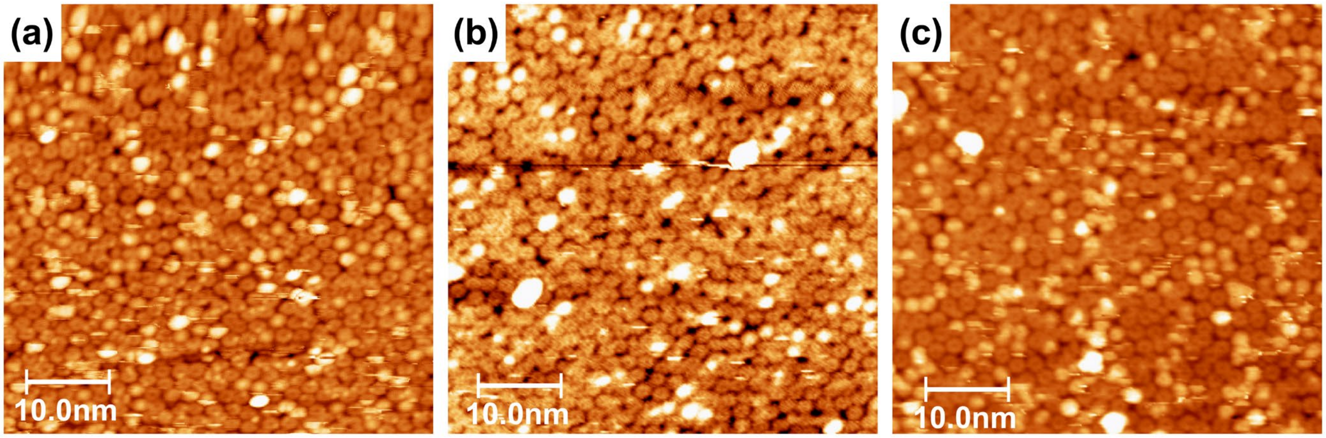

Figure S5. Additional STM images of high coverage samples annealed to 653 K with similar appearance from Figure 3i. (a-c) different high coverage (full layer) samples after annealing to 653 K. The appearance of bowl up/down molecules representing **ZnTAP–24H** is consistent with one shown in Figure 3i.

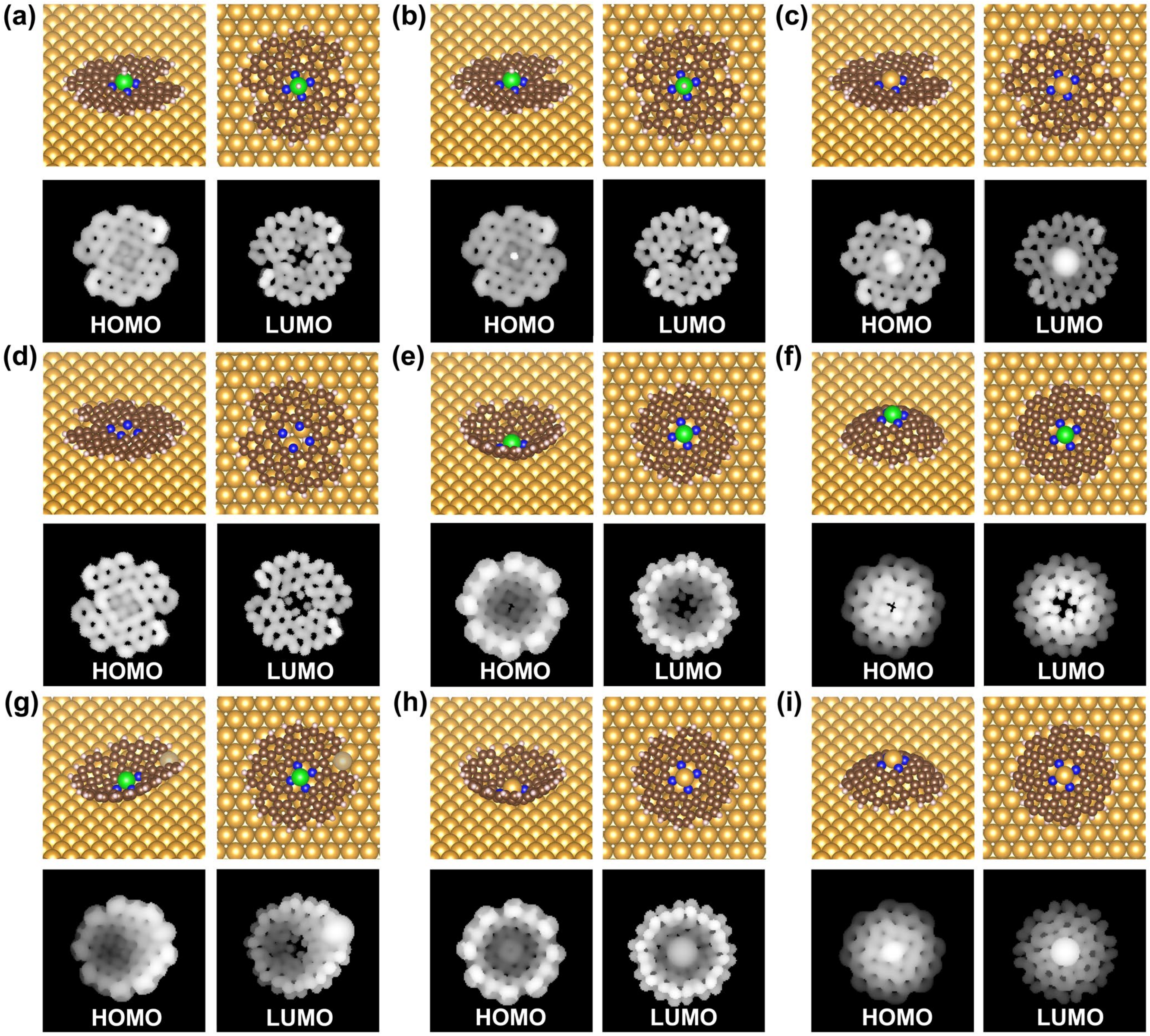

Figure S6. Comparison of optimized structures and occupied/unoccupied states density maps of several ZnTAP–20H derived models in order to match experimental appearance from Figure 3i. (a) native **ZnTAP–20H** molecule with hydrogen molecule adsorbed onto Zn in an upright orientation. (b) native **ZnTAP–20H** molecule with hydrogen atom adsorbed onto Zn. (c) native **ZnTAP–20H** molecule transmetalated with Au (**AuTAP–20H**). (d) demetalated native **ZnTAP–20H** molecule (**2HTAP–20H**). (e) **ZnTAP–24H** molecule adsorbed in a bowl-up configuration. (f) **ZnTAP -24H** molecule adsorbed in a bowl-down configuration. (g) **ZnTAP–22H** molecule (with Au adatom) adsorbed in a bowl-up configuration. (h) **AuTAP–24H** molecule adsorbed in a bowl-up configuration. (i) **AuTAP–24H** molecule adsorbed in a bowl-down configuration. The **ZnTAP–24H** molecule adsorbed in a bowl-up/down configurations from (e) and (f) as well as **ZnTAP–22H** molecule (with Au adatom) from (g) match the best appearance of the molecules shown in Figure 3i.

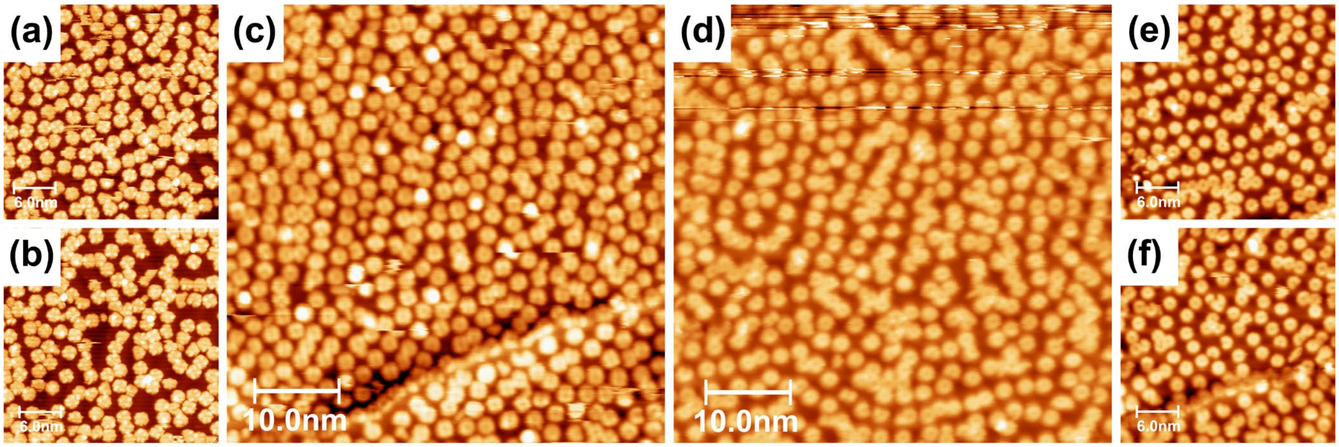

Figure S7. STM images upon annealing sequence of a sample with intermediate coverage (~65 %) (a-c) three STM images of the intermediate coverage (~65 %) sample after annealing to 603 K. The appearance of molecules representing **ZnTAP–16H** is consistent with the appearances in Figure 3c and Figure S3. (d-f) three STM images of the intermediate coverage (~65 %) sample after annealing to 603 K. The appearance of individual molecules representing **ZnTAP–20H** is consistent with the appearance in Figure 3e, however oligomers like the ones from Figure 3f-h area already present.

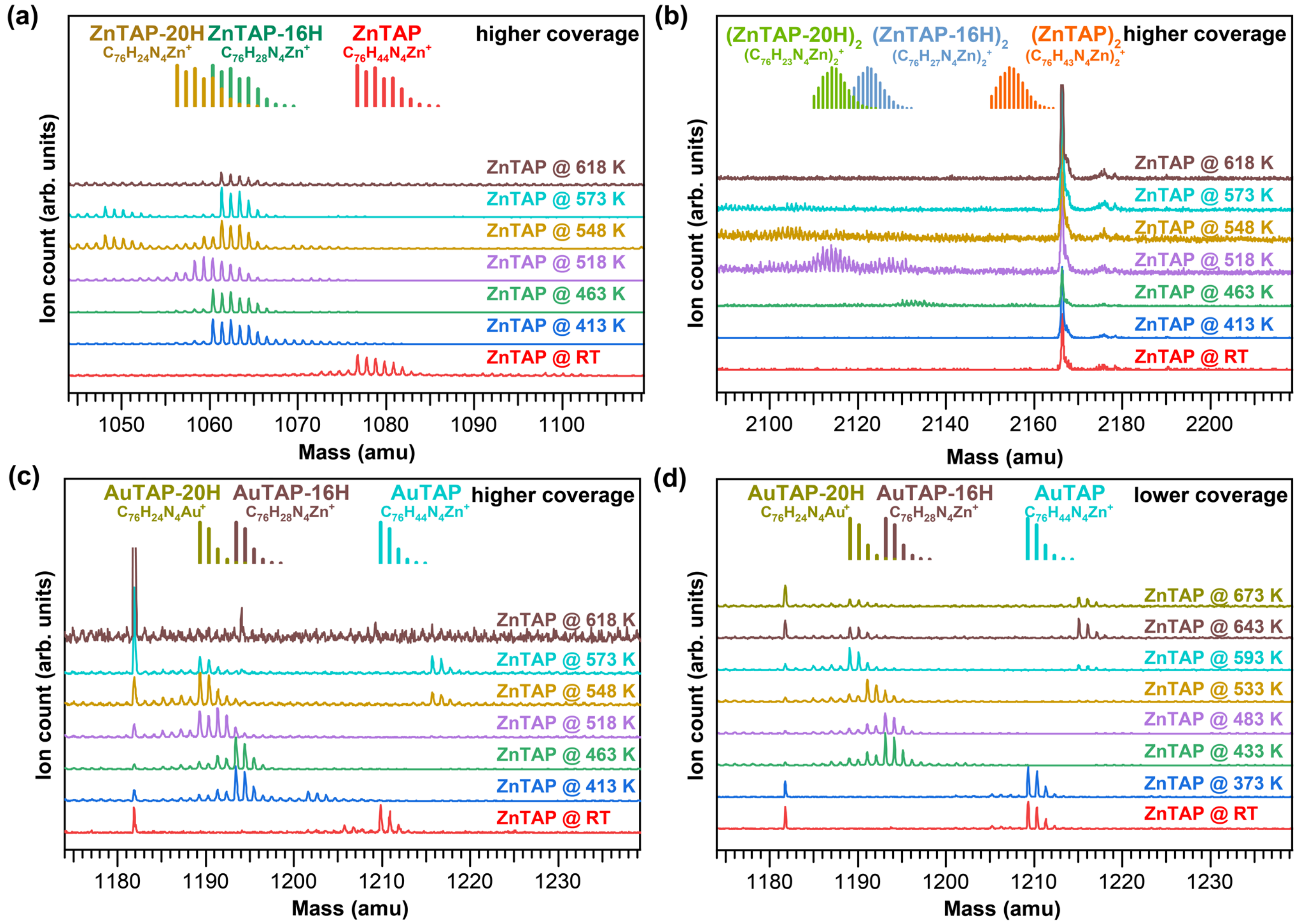

Figure S8. Different mass regions of ToF-SIMS tracking molecular cyclodehydrogenation and transmetallation (a) ToF-SIMS temperature series of higher coverage **ZnTAP** on Au(111) showing masses corresponding to **ZnTAP** and **ZnTAP–16H**. Notably, **ZnTAP–20H** are not detected and specific signals originating from **ZnTAP** are lost already at 548 K. (b) ToF-SIMS temperature series of the same higher coverage **ZnTAP** sample of the **ZnTAP, ZnTAP–16H** and **ZnTAP–20H** dimers. (c,d) ToF-SIMS temperature series of **AuTAP, AuTAP–16H** and **AuTAP–20H** of the same higher coverage **ZnTAP** sample (c) and of the lower coverage sample shown in Figure 1b (d). Simulated mass distributions thereof are shown above the spectra. For the dimer spectra, formation of one C-C bond between the monomers is assumed.

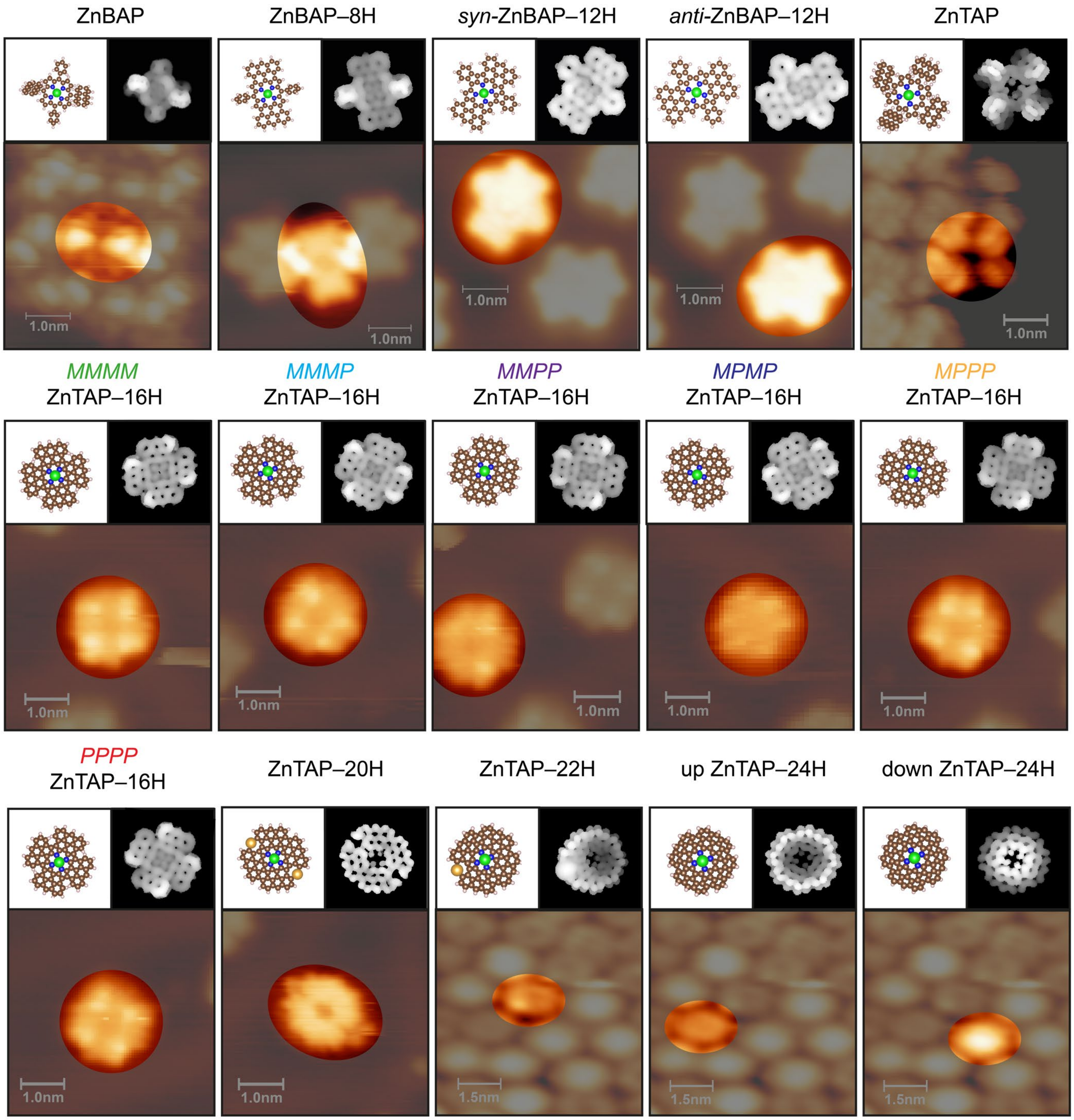

Figure S9. Overview of the observed surface products in Figure 2 and Figure 3 (Figure S3b) Molecular structures, simulated state density maps and close up STM images of the observed **ZnBAP/ZnTAP** related species and surface products. Note that products missing one anthracenyl unit, transmetallated products or **ZnTAP–20H** oligomers are not shown. The STM images are extracts from Figure 2 (**ZnBAP** related species) and Figure 3 (**ZnTAP** related species) as well as Figure S3b (*MMPP* and *MPMP* **ZnTAP–20H** species)

Table S3. STM imaging parameters

Figure	Temperature [K]	Size [nm²]	U [V]	I [pA]
Figure 2a	7	25 x 25	1.75	50
Figure 2d	7	30 x 30	-1.75	1000
Figure 2g	7	30 x 30	-1.75	110
Figure 3a	50	15 x 15	1.25	50
Figure 3c	7	30 x 30	-2.20	540
Figure 3e	7	19.6 x 19.6	1.50	100
Figure 3f	7	50 x 50	1.75	50
Figure 3g	7	15.3 x 15.3	0.75	1000
Figure 3h	7	15.3 x 15.3	0.75	1000
Figure 3i	50	50 x 33.2	2.20	50
Figure S1a	7	30 x 30	-1.75	110
Figure S1b	7	30 x 30	-1.75	110
Figure S1c	7	30 x 30	-1.75	110
Figure S2a	50	500 x 500	2.15	50
Figure S2b	50	200 x 200	-2.15	100
Figure S3a	7	50 x 50	-2.20	100
Figure S3b	7	100 x 100	-2.20	100
Figure S7a	50	50 x 50	1.00	50
Figure S7b	50	50 x 50	2.20	50
Figure S7c	50	50 x 50	2.20	50
Figure S8a	7	30 x 30	-1.75	100
Figure S8b	7	30 x 30	-2.20	100
Figure S8c	7	50 x 50	2.20	50
Figure S8d	7	50 x 50	2.20	50
Figure S8e	7	30 x 30	1.25	1000
Figure S8f	7	30 x 30	1.25	1000

REFERENCES

(1) Sooambar, C.; Troiani, V.; Bruno, C.; Marcaccio, M.; Paolucci, F.; Listorti, A.; Belbakra, A.; Armaroli, N.; Magistrato, A.; Zorzi, R. D.; Geremia, S.; Bonifazi, D. Synthesis, Photophysical, Electrochemical, and Electrochemiluminescent Properties of 5,15-Bis(9-Anthracenyl)Porphyrin Derivatives. *Org. Biomol. Chem.* **2009**, *7*, 2402–2413.

(2) Bonifazi, D.; Accorsi, G.; Armaroli, N.; Song, F.; Palkar, A.; Echegoyen, L.; Scholl, M.; Seiler, P.; Jaun, B.; Diederich, F. Oligoporphyrin Arrays Conjugated to [60]Fullerene: Preparation, NMR Analysis, and Photophysical and Electrochemical Properties. *Helv. Chim. Acta* **2005**, *88*, 1839–1884.

(3) Volz, H.; Schäffer, H. Mesosubstituted Porphyrins. III. 5,10,15,20-Tetraanthracenylporphyrin. *Chem. Ztg* **1985**, *109*, 308–309.

(4) Rousseau, K.; Dolphin, D. A Purification of *Meso*-Tetraphenylporphyrin. *Tetrahedron Lett.* **1974**, *15*, 4251–4254.
